# Coreduction methodology for immiscible alloys of CuRu solid-solution nanoparticles with high thermal stability and versatile exhaust purification ability†

**DOI:** 10.1039/d0sc03373a

**Published:** 2020-09-18

**Authors:** Bo Huang, Hirokazu Kobayashi, Tomokazu Yamamoto, Syo Matsumura, Yoshihide Nishida, Katsutoshi Sato, Katsutoshi Nagaoka, Masaaki Haneda, Shogo Kawaguchi, Yoshiki Kubota, Hiroshi Kitagawa

**Affiliations:** Division of Chemistry, Graduate School of Science, Kyoto University Kitashirakawa-Oiwakecho, Sakyo-ku Kyoto 606-8502 Japan kitagawa@kuchem.kyoto-u.ac.jp bohuang@xjtu.edu.cn; JST, PRESTO 4-1-8 Honcho Kawaguchi Saitama 332-0012 Japan; Department of Applied Quantum Physics and Nuclear Engineering, Kyushu University Motooka 744, Nishi-ku Fukuoka 819-0395 Japan; The Ultramicroscopy Research Centre, Kyushu University Motooka 744, Nishi-ku Fukuoka 819-0395 Japan; INAMORI Frontier Research Centre, Kyushu University Motooka 744, Nishi-ku Fukuoka 819-0395 Japan; Department of Chemical Systems Engineering, Graduate School of Engineering, Nagoya University Furo-cho, Chikusa-ku Nagoya 464-8603 Japan; Elements Strategy Initiative for Catalysts and Batteries, Kyoto University 1-30 Goryo-Ohara, Nishikyo-ku Kyoto 615-8245 Japan; Advanced Ceramics Research Centre, Nagoya Institute of Technology 10-6-29 Asahigaoka, Tajimi Gifu 507-0071 Japan; Frontier Research Institute for Materials Science, Nagoya Institute of Technology Gokiso-cho, Showa-ku Nagoya 465-8555 Japan; Japan Synchrotron Radiation Research Institute (JASRI) SPring-8, 1-1-1 Kouto, Sayo-cho, Sayo-gun Hyogo 679-5198 Japan; Department of Physical Science, Graduate School of Science, Osaka Prefecture University Sakai Osaka 599-8531 Japan; Institute for Integrated Cell-Material Sciences (iCeMS), Kyoto University Yoshida, Sakyo-ku Kyoto 606-8501 Japan

## Abstract

This study provides a coreduction methodology for solid solution formation in immiscible systems, with an example of a whole-region immiscible Cu–Ru system. Although the binary Cu–Ru alloy system is very unstable in the bulk state, the atomic-level well-mixed CuRu solid solution nanoparticles were found to have high thermal stability up to at least 773 K in a vacuum. The exhaust purification activity of the CuRu solid solution was comparable to that of face-centred cubic Ru nanoparticles. According to *in situ* infrared measurements, stronger NO adsorption and higher intrinsic reactivity of the Ru site on the CuRu surface than that of a pure Ru surface were found, affected by atomic-level Cu substitution. Furthermore, CuRu solid solution was a versatile catalyst for purification of all exhaust gases at a stoichiometric oxygen concentration.

## Abstract 2

This study provides a coreduction methodology for solid solution formation in immiscible systems, with an example of a whole-region immiscible Cu–Ru system.

## Graphical abstract

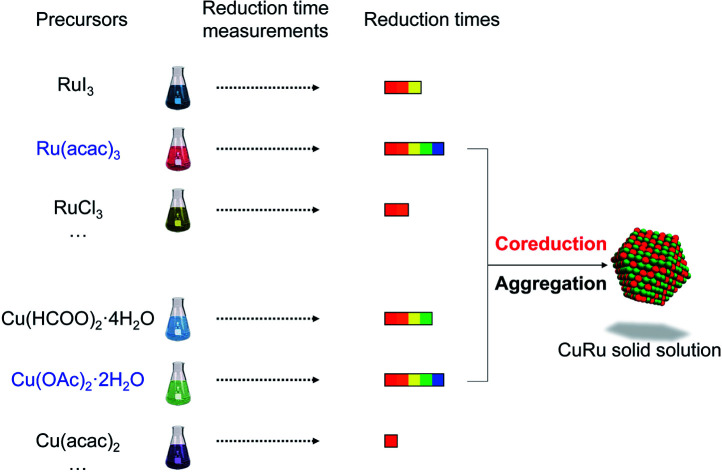

## Introduction

Platinum group metals (PGMs) play a crucial role in both industrial and scientific areas, including use in fuel cells,^1,2^ water splitting^3,4^ and exhaust purification.^5,6^ Due to their extremely low reserves in the Earth's crust, decreasing the demand for PGMs is extremely important. Alloying PGMs with earth-abundant metals (EAMs) has attracted much attention to decrease the usage of PGMs, and to simultaneously generate synergic performance in specific systems.^7,8^ Of all the alloy types, solid solutions are the most effective at controlling the electronic structure, which leads to better performance in applications such as catalysis,^9,10^ optics^11,12^ and magnetism.^13,14^ However, for the most famous catalytic reaction of PGMs – exhaust purification – there has been little research on the catalytic ability of solid solutions of PGMs alloyed with EAMs as exhaust purification catalysts. This may be due to the decreased exhaust purification activity or poor thermal stability of PGMs-EAMs solid solutions prepared to date. In fact, the number of available PGMs-EAMs solid solutions has been highly restricted due to the solubility of different metals in binary systems; in many binary systems two different metal elements cannot be mixed (known as immiscible systems). Among these immiscible binary systems, Cu–Ru is one of the most challenging systems since Cu and Ru elements are completely immiscible at any temperature and composition in the bulk state, including the liquid phase.^15^ Up to now, much effort has been made for preparation of CuRu nanoparticles in colloidal chemistry or theoretical calculations.^16–19^ Breaking through this limitation in solid solution formation for immiscible systems is highly important, especially for the development of novel exhaust purification catalysts with PGMs and EAMs. Until now, there has been no practical methodology reported for immiscible binary systems, although there are a few successful syntheses of bimetallic solid solutions.^20–25^

In this work, the first methodology for solid solution formation in an immiscible binary system is reported, with the example of a whole-region immiscible Cu–Ru system. This methodology was proved to be very practical for investigating the coreduction conditions and other parameters; the coreduction synthesized CuRu solid-solution nanoparticles (NPs) had an atomic-level randomly mixed alloying state, and were confirmed to have high thermal stability up to 773 K, suitable for low-temperature exhaust purification reactions.^26^

## Results and discussion

### Coreduction methodology

**Fig. 1:**
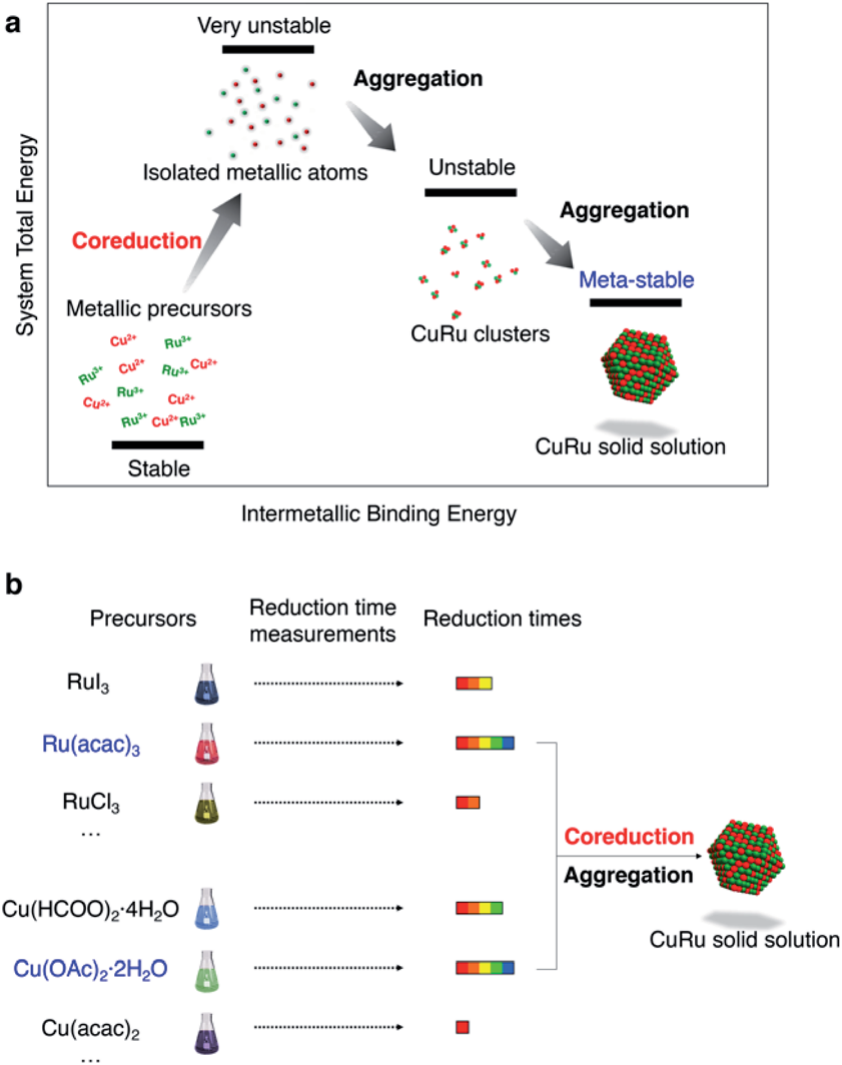
Schematic images for the coreduction mechanism and coreduction methodology. Schematic of the (a) coreduction mechanism for solid solution structure formation and the (b) methodology to achieve coreduction conditions.

Coreduction is considered necessary for solid-solution structure formation in immiscible systems, where two different metallic precursors are reduced simultaneously to isolated atoms, followed by aggregation to clusters and ending up with a solid solution (Fig. 1a). Unlike the rapid synthetic strategy achieved by using strong reductants,^27^ this methodology involved searching for coreduction conditions by measuring the reaction times of several potential precursors to find a combination that had similar reaction times to each other (Fig. 1b).

**Fig. 2:**
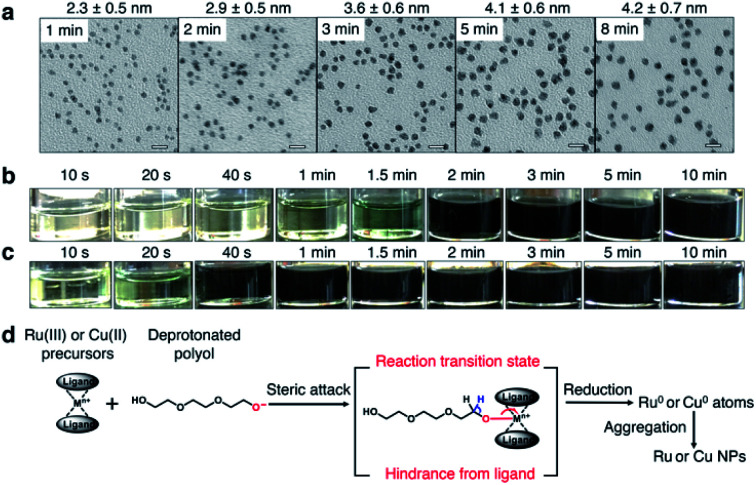
Reaction time measurements and reaction mechanism. Reaction time measurement results of (a) Ru(acac)_3_ by TEM measurements, and (b) Cu(OAc)_2_·H_2_O and (c) Cu(HCOO)_2_·4H_2_O by colorimetry. All scale bars in (a) are 10 nm. (d) Proposed reaction intermediate showing attack on the ligand-protected metallic ion centre by a polyol molecule.

The Ru precursor was determined to be ruthenium acetylacetonate, Ru(acac)_3_, since it can form face-centred cubic (fcc)-Ru NPs^28^ and is easier to mix with fcc-structured Cu according to the Hume-Rothery rules.^29^ The reaction time was measured for Ru(acac)_3_ by sampling the reaction solution at several times during a typical polyol reduction reaction (see the ESI and Fig. S1†). The aliquots were analysed by transmission electron microscopy (TEM), as shown in Fig. 2a. A continuous increase in the mean diameter of fcc-Ru NPs was observed from 1–5 min, and after 5 min the mean diameter remained constant; therefore, the reaction time of Ru(acac)_3_ was considered to be *ca.* 5 min.

For Cu, three potential precursors were considered: copper acetylacetonate, Cu(acac)_2_, copper acetate monohydrate Cu(OAc)_2_·H_2_O and copper formate tetrahydrate Cu(HCOO)_2_·4H_2_O. The reaction times of these Cu precursors were measured by the same method as for Ru(acac)_3_. The reaction time of Cu(acac)_2_ was determined by TEM analysis to be *ca.* 1 min (Fig. S2†). The reaction time of Cu(OAc)_2_·H_2_O was determined by colorimetry to be *ca.* 5 min (Fig. 2b), which was the same as that of Ru(acac)_3_. The reaction time of Cu(HCOO)_2_·4H_2_O was *ca.* 2 min (Fig. 2c). It was proposed that the reduction of metallic precursors to metallic nanoparticles by polyol molecules involved a reaction intermediate, in which the bulky ligands sterically impeded the attack of polyol molecules on the ligand-protected metallic ion centre, thereby blocking the formation of oxygen–metal bonds (Fig. 2d). As shown in Fig. S3,† Ru(iii) in Ru(acac)_3_ is at the centre of a hexa-coordinate arrangement with large ligands, which slowed down the steric attack process. This was consistent with the relatively long reaction time of *ca.* 5 min shown in Fig. 2a.

Similarly, Cu(ii) is in a penta-coordinate dimeric structure in Cu(OAc)_2_·H_2_O, which is very similar to a hexa-coordinate structure with large hindrance. Considering the similar redox potentials of Cu^2+^ and Ru^3+^ (Table S1†), the redox potential effect of this Cu–Ru system contributed little to the velocity difference of the Cu^2+^ and Ru^3+^ ion centres. Thus, a similar reaction time of Cu(OAc)_2_·H_2_O and Ru(acac)_3_ resulted, *ca.* 5 min. On the other hand, Cu(ii) at the centre of tetra-coordinated Cu(HCOO)_2_·4H_2_O and tetra-coordinated Cu(acac)_2_ with small hindrance gave relatively short reaction times of *ca.* 2 min and *ca.* 1 min, respectively. The faster reaction velocity of Cu(acac)_2_ than Cu(HCOO)_2_·4H_2_O may have arisen from electron donation by the methyl group in the acetylacetonate ligand to the Cu(ii) ion centre *via* a conjugation structure.

After measuring the reaction times of each precursor, the coreduction conditions for CuRu alloy synthesis were experimentally tested by the reaction of each combination using Ru(acac)_3_ with each of Cu(acac)_2_, Cu(HCOO)_2_·4H_2_O and Cu(OAc)_2_·H_2_O. For the combination of Ru(acac)_3_ and Cu(acac)_2_, the reaction product showed an asymmetric powder X-ray diffraction (PXRD) pattern containing a large amount of the Cu pattern (Fig. S4†). Core-shell structured NPs accompanying Ru NPs were also found by scanning transmission electron microscopy-energy-dispersive X-ray spectroscopy (STEM-EDX) mapping (Fig. S5†). This core–shell structure formation demonstrated a high reaction velocity difference between Ru(acac)_3_ and Cu(acac)_2_ (Fig. S6†), which was consistent with the reaction time measurement results. For the combination of Ru(acac)_3_ and Cu(HCOO)_2_·4H_2_O, the PXRD pattern of the product was much better than that of Cu(acac)_2_, but the by-product was still included as indicated by the asymmetric broad main peak at a 2*θ* of 42° (Fig. S7 and Table S2†). The multiple-component product observed by TEM (Fig. S8†) was a result of the different reaction times of Ru(acac)_3_ and Cu(HCOO)_2_·4H_2_O. For the combination of Ru(acac)_3_ and Cu(OAc)_2_·H_2_O, a symmetrical PXRD pattern and single-size distribution in the TEM image were obtained, which demonstrated the formation of a single component (Figs. S9, S10 and Table S3†). Furthermore, the random distribution of Cu and Ru elements was directly observed by conducting STEM-EDX mapping measurements (Fig. S11†). However, a lower Cu content was found: *ca.* 25 at% compared to the nominal ratio of 50 at% (Fig. S12†). This random alloy structure showed good consistency with coreduction conditions in which the reaction times of Ru(acac)_3_ and Cu(OAc)_2_·H_2_O are the same. Notably, the coreduction conditions were interrupted at exactly the same condition except that anhydrous solvent was used, and the product was estimated to be a mixture of fcc-Ru and Cu components (Fig. S13, S14 and Table S4†). The increased reaction velocity from Cu(ii) to Cu(0) by using the anhydrous solvent is considered to be due to the removal of coordinating H_2_O in Cu(OAc)_2_·H_2_O dimers, resulting in its change from penta-coordinate to tetra-coordinate.

To further optimize the coreduction conditions, based on conditions for the combination of Ru(acac)_3_ and Cu(OAc)_2_·H_2_O, the following factors were also investigated: temperature (Figs. S15, S16 and Table S5†); atmosphere (Fig. S17, S18 and Table S6†); reducing agents and solvent (Fig. S19–S23, Scheme S1, Tables S7 and S8†); solvent oxygen content (Fig. S24–S26 and Table S9†), by 3 days of degassing with liquid N_2_; and stirring speed (Fig. S27–S30 and Table S10†). Based on their impact on the reaction, the relative importance of these factors for the Cu–Ru system was estimated as follows: precursor > reducing agent and solvent > solvent oxygen content > stirring speed > atmosphere > temperature. Specifically, for the Cu(OAc)_2_·H_2_O precursor, the anhydrous solvent was the most important factor. Finally, the coreduction conditions were determined to be the same as those of a previously reported protocol^16^ and finally achieved 46.3 at% Cu content close to the nominal ratio, determined by X-ray fluorescence (XRF) measurements (Table S11†). The CuRu NPs of this study were obtained under the same synthetic conditions.

In addition to PXRD, TEM, STEM-EDX mapping and XRF results, high resolution STEM and X-ray photoelectron spectroscopy (XPS) measurements were performed to further characterize the obtained CuRu NPs. From the high resolution STEM image of the CuRu NPs, the lattice fringe distance was determined to be 2.16 Å, which is consistent with the {111} lattice plane calculated for an fcc-Cu_0.5_Ru_0.5_ alloy (Fig. S31†). The electronic state change due to the homogeneous alloy formation by XPS measurements is shown in Fig. S32 and Table S12.† The Ru 3p binding energy of the CuRu NPs shifted to higher energy with Cu alloying, which is in agreement with the Ru electron deficiency. Meanwhile, the Cu 2p binding energy of the CuRu NPs shifted to lower energy with Ru alloying, which is in agreement with electron filling-in of Cu. Therefore, the XPS spectra indicate the electronic state difference for CuRu NPs compared with those of Cu and Ru metals, which provides further proof for the atomic-level surface homogeneity of Cu and Ru by the coreduction methodology. Combining the above characterization, the CuRu solid solution NPs were successfully synthesized where Cu and Ru were homogeneously mixed at the atomic level. Since Cu and Ru are totally immiscible in any region of the bulk-state phase diagram,^15^ even in the liquid phase, the successful atomic-level mixing of this challenging Cu–Ru combination strongly suggested that the methodology employed in this study for the determination of coreduction conditions for solid solution formation could be universal for immiscible systems.

### Thermal stability

**Fig. 3:**
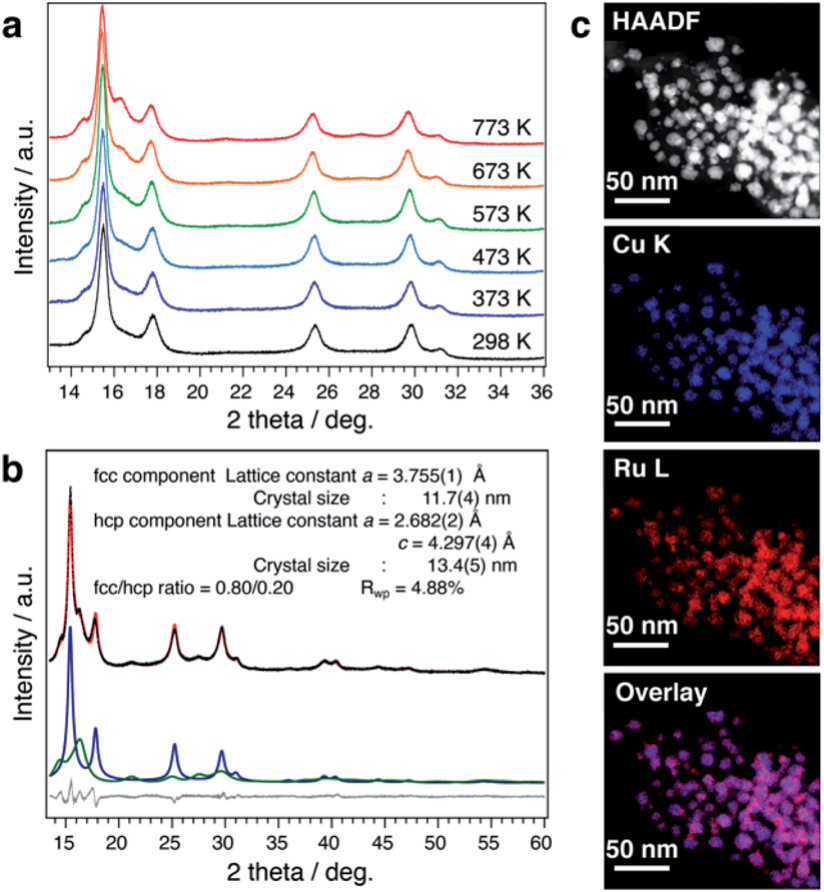
Thermal stability of CuRu solid-solution nanoparticles. (a) *In situ* synchrotron PXRD measurements under vacuum from 298–773 K with 0.5787 Å wavelength. (b) The synchrotron XRD pattern of CuRu nanoparticles (black dots) at 773 K and the calculated pattern (red line). The bottom lines show the difference profile (grey) and the fitted curves of the fcc component (blue) and hcp component (green). (c) High-angle annular dark-field (HAADF)-STEM, Cu–K, Ru–L and reconstructed overlay STEM-EDX maps obtained from a group of CuRu NPs after heating at 773 K. All scale bars are 50 nm.

It is well known that the solid-solution state of immiscible systems is thermodynamically unstable in the bulk state due to the tremendous mixing enthalpy from the large local lattice strain caused by the large difference of atomic radii, known as the Hume-Rothery rules (Fig. S33A†).^29^ Although the unstable solid-solution state of immiscible systems can be stabilized by increasing mixing entropy, through the formation of high entropy alloys,^30^ it can only be stabilized when the number of elements is more than four. Recently, some new findings have suggested that the surface of nanoscale materials has a much more flexible structure.^31^ The possible mechanism for stabilization of the solid-solution state on the nanoscale is considered to be relaxation of local strain by the more flexible structure of nanoparticles, as compared to with the rigidity of bulk material structures (Fig. S33B†). This prompted us to investigate the thermal stability of CuRu solid-solution NPs, even though the bulk state solid-solution Cu–Ru system is very unstable.^15,32^ The thermal stability of CuRu solid-solution NPs was determined by *in situ* synchrotron XRD in capillary under vacuum. Surprisingly, no phase separation was observed from 298 K up to 773 K (Fig. 3a). Rietveld refinement results also suggested the solid-solution structure of each component at 773 K, with only a small ratio of fcc-to-hcp (hexagonal close-packed) phase transition (Fig. 3b and Table S13†). Furthermore, STEM-EDX mapping measurements were performed on the sample after the 773 K thermal stability, revealing homogenous distribution of Cu and Ru elements and providing evidence of the high thermal stability of CuRu solid-solution NPs up to at least 773 K in a vacuum (Fig. 3c and Fig. S34†). It was proposed that an atomic-level well mixed solid solution may have higher stability than that of a domain mixed alloy, since domain mixing alloy is the intermediate state before conversion to the phase separation alloy, by sufficient heating (Fig. S35A†). It is believed that the high thermal stability of CuRu solid-solution NPs originated from the well optimized coreduction conditions, which led to a solid-solution structure that was homogeneously mixed at the atomic level (Fig. S35B†).

### Exhaust purification activity

PGMs are well known as practical catalysts for exhaust purification reactions in the automotive industry, and replacement of PGMs to reduce the cost has long been a key issue. Cu is an earth-abundant metal with a much lower price and higher natural reserves; The high thermal stability of the CuRu solid-solution NPs formed in this study inspired the application of the solid solution to an exhaust purification test. Fcc-Ru NPs were also synthesized as a comparison.

**Fig. 4:**
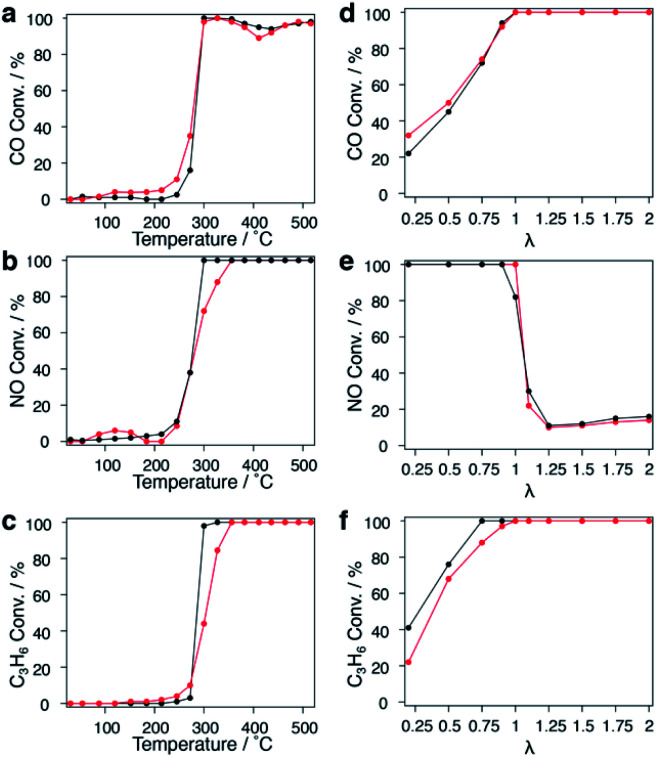
Exhaust purification performance of CuRu solid-solution nanoparticles. Temperature dependence of (a) CO, (b) NO, and (c) C_3_H_6_ conversion in exhaust purification reactions for CuRu/γ-Al_2_O_3_ (red), and fcc-Ru/γ-Al_2_O_3_ (black) at *λ* = 1. *λ* dependence of (d) CO, (e) NO, and (f) C_3_H_6_ conversion for CuRu/γ-Al_2_O_3_ (red), and fcc-Ru/γ-Al_2_O_3_ (black) at 400 °C.

The extra polyvinylpyrrolidone (PVP) of CuRu and fcc-Ru NPs was removed by repeated ethanol washing down to 34.4 and 38.8 wt%, respectively; the remaining PVP will be decomposed during the catalytic test.^33^ CuRu solid-solution and fcc-Ru NPs were supported on γ-Al_2_O_3_ at 1 wt% *via* the wet impregnation method (see the ESI†). The HAADF-STEM image of a γ-Al_2_O_3_ supported nanoalloy exhibits good dispersion of CuRu NPs (Fig. S36†). The exhaust purification tests for each catalyst were carried out from r.t. to 773 K without further pre-treatment, at a stoichiometric O_2_ concentration of *λ* = 1 (see the ESI†). The catalytic activities expressed as *T*_50_ of CuRu solid-solution NPs and fcc-Ru NPs were almost identical for NO_*x*_ reduction and CO oxidation, even though 50 at% of the PGM Ru element was replaced by inexpensive Cu (Fig. 4a and b). Meanwhile, the catalytic activity of CuRu solid-solution NPs for C_3_H_6_ oxidation was close to that of fcc-Ru NPs (Fig. 4c).

The *λ* dependence of each catalyst at a fixed temperature of 400 °C was also investigated. The activity of CuRu solid-solution NPs for CO oxidation was slightly higher than that of fcc-Ru NPs within *λ* < 1 (Fig. 4d), while no obvious difference was found between CuRu solid-solution and fcc-Ru NP activities for NO_*x*_ reduction within a wide *λ* range, except for *λ* = 1 (Fig. 4e). CuRu solid-solution NPs performed moderately in C_3_H_6_ oxidation compared with fcc-Ru NPs, within *λ* < 1 (Fig. 4f). Interestingly, in the range near *λ* = 1, all the CO, NO and C_3_H_6_ conversions of CuRu solid-solution NPs reached 100%, and performed better than fcc-Ru NPs. The versatile CuRu solid solution catalyst has great potential to be applied as a single-component exhaust purification catalyst. Even Rh, the most famous exhaust purification catalyst, cannot simultaneously purify CO, NO_*x*_ and hydrocarbon species up to 100%, it also requires Pt and Pd as co-catalysts for complete CO conversion.^34^ Notably, under the conditions of the similar loading amount and the same alumina support, the CuRu solid solution catalyst exhibited close conversion with Pd/Rh and Pt/Rh catalysts in NO reduction and hydrocarbon oxidation, but did not match with Pd/Rh and Pt/Rh catalysts in CO conversion;^35^ this may be due to a relatively large mean diameter for CuRu NPs (Fig. S37, S38†). Interestingly, N_2_O (damaging the central nervous system^36^ and ozonosphere^37^) was not detected from the CuRu catalysed reaction by gas chromatography (GC); however, Pt/Rh and Pt/Rh catalysts generated much N_2_O in a lower temperature range.^35^ Furthermore, considering that the mean particle size of CuRu solid solution is larger than that of fcc-Ru NPs (Fig. S37†), the intrinsic exhaust purification activity of CuRu solid solution is higher than that of fcc-Ru NPs. The mass and heat transfer could be neglected according to the calculations (see the Experimental details in the ESI†). Finally, repetitive tests showed that the NO_x_ reduction ability decreased especially after the second test (Fig. S39†).

**Fig. 5:**
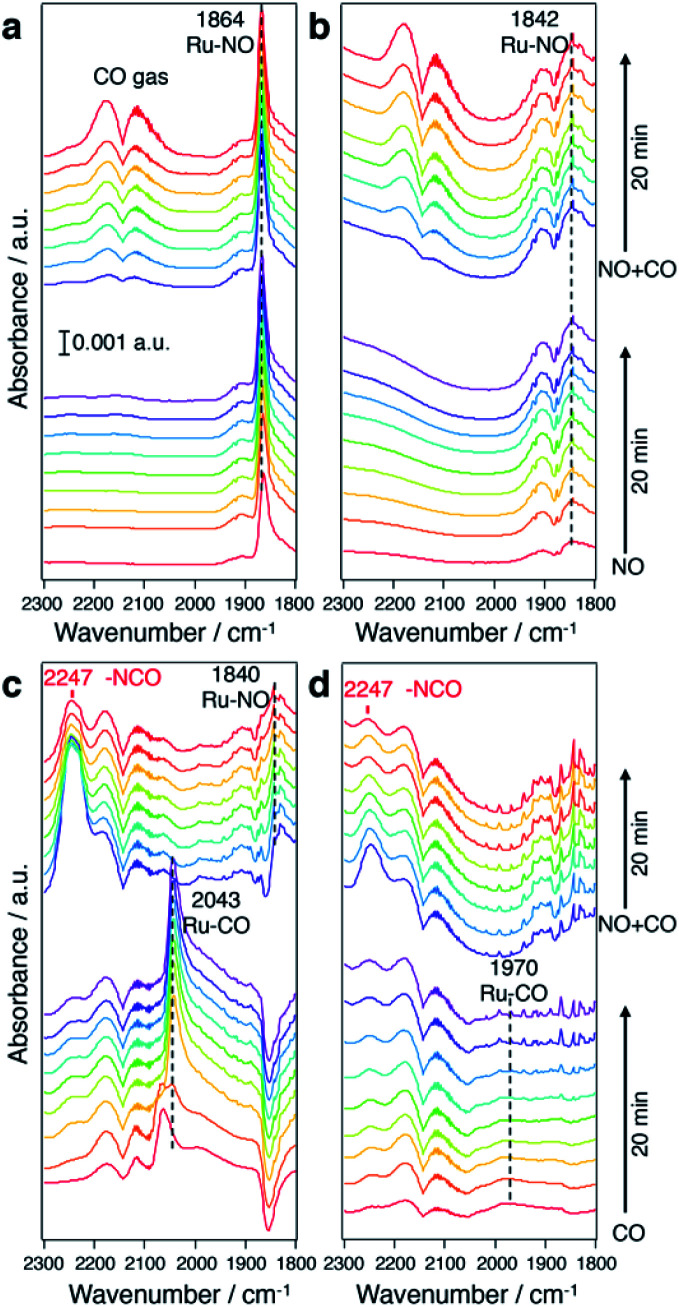
*In situ* FTIR spectra under a NO and CO flow. *In situ* FTIR spectra of (a) CuRu/γ-Al_2_O_3_ and (b) fcc-Ru/γ-Al_2_O_3_ under a NO flow followed with a NO + CO flow. *In situ* FTIR spectra of (c) CuRu/γ-Al_2_O_3_ and (d) fcc-Ru/γ-Al_2_O_3_ under a CO flow followed with a CO + NO flow. All the spectra were measured at 200 °C with He balance.

To understand the origin of the higher intrinsic exhaust purification activity of CuRu solid solution, *in situ* Fourier transform infrared spectroscopy (FTIR) was performed (Fig. 5). In Fig. 5a and b, under a NO flow, a much stronger NO adsorption onto Ru sites (at 1864 cm^−1^) was found for CuRu compared with fcc-Ru, and disabled CO adsorption during a NO + CO flow. In Fig. 5c and d, under a CO flow, an intense IR band at 2043 cm^−1^ due to CO adsorbed on Ru was detected in CuRu. This proved its better adsorption ability for CO than what was found for fcc-Ru. With the addition of NO, the Ru–CO species was quickly consumed, with the formation of N=C=O species with a shoulder peak at 2247 cm^−1^,^38^ which may act as the intermediate of NO_x_ reduction with CO and then finally be decomposed to N_2_ and CO_2_.^39^ Thus, the higher intrinsic exhaust purification activity of CuRu solid solution may originate from the intensive interaction between reaction molecules and the isolated highly active Ru sites under Cu atomic-level substitution. Meanwhile, considering that the Cu element is easily oxidized under reaction condition Cu sites were found to be intrinsically inert to NO_*x*_ reduction in CuRu. In addition, the *in situ* FTIR results revealed that no N_2_O species (sharp double peaks at 2237 and 2210 cm^−1^)^40^ was detected for the CuRu catalyst, consistent with GC analyses of catalytic products.

## Conclusions

This study provides a methodology for achieving the optimal coreduction conditions of the metallic precursors, investigated from the viewpoint of the steric hindrance effect on the reaction times of the metallic precursors. By applying this methodology, atomic-level homogeneous mixing was successful even in the whole-region immiscible Cu–Ru system. This well-mixed CuRu solid solution demonstrated high thermal stability up to at least 773 K in a vacuum. For this CuRu solid-solution three-way catalyst, even though 50% of the PGM Ru was replaced by EAM Cu, its exhaust purification activity was still comparable to that of fcc-Ru NPs. Furthermore, the CuRu solid solution performed well with versatile exhaust purification ability and zero N_2_O emission, thus promising to be an alternative exhaust purification catalyst for further application in the automotive industry. Most importantly, the coreduction methodology presented in this report will greatly shorten the synthetic trial and error period for new solid solutions in other immiscible systems, and give much valuable insight into functional materials developments.

## Conflicts of interest

There are no conflicts to declare.

## Supplementary Material

SC-011-D0SC03373A-s001
