# Supplementary material for: Coreduction methodology for immiscible alloys of CuRu solid-solution nanoparticles with high thermal stability and versatile exhaust purification ability
Source: Chem Sci. 2020 Sep 18;11(42):11413–8. doi: 10.1039/d0sc03373a (PMC8162931; doi:10.1039/d0sc03373a)
Supplement: SC-011-D0SC03373A-s001 [file SC-011-D0SC03373A-s001.pdf]

Electronic Supplementary Material (ESI) for Chemical Science.
This journal is © The Royal Society of Chemistry 2020

Supplementary Information

Coreduction Methodology for Immiscible Alloy of CuRu Solid-Solution Nanoparticles with High Thermal Stability and Versatile Exhaust Purification Ability

Bo Huang,* Hirokazu Kobayashi, Tomokazu Yamamoto, Syo Matsumura, Yoshihide Nishida, Katsutoshi Sato, Katsutoshi Nagaoka, Masaaki Haneda, Shogo Kawaguchi, Yoshiki Kubota and Hiroshi Kitagawa*

*Correspondence and requests for materials should be addressed to

H. Kitagawa (email: kitagawa@kuchem.kyoto-u.ac.jp) and B. Huang (email: bohuang@xjtu.edu.cn).

1. Experimental details

Transmission electron microscopy (TEM) images: TEM images were captured using a Hitachi HT7700 operated at 100 kV accelerating voltage.

High-resolution Scanning TEM (HR-STEM) images and STEM- Energy Dispersive X-ray spectrometry (EDX) mapping images: HR-STEM images and STEM-EDX mapping images were captured using a JEOL ARM 200F STEM instrument operated at 200 kV accelerating voltage.

X-ray fluorescence (XRF) measurements: XRF measurements were performed using a Rigaku ZSX Primus IV.

X-ray diffraction (XRD) measurements: The crystal structures were investigated by powder XRD analysis using a Bruker D8 Advance diffractometer (Cu $K\alpha$ radiation).

Synchrotron XRD and in situ XRD measurements: The crystal structures were investigated by capillary synchrotron XRD analysis measured at the BL02B2 beamline, SPring-8. The XRD patterns of the samples sealed in a glass capillary was measured in situ with a wavelength of 0.5787 Å. The thermal stability of $Cu_{0.5}Ru_{0.5}$ NPs was investigated by in situ powder XRD analysis measured at the same beamline under vacuum in the temperature range between 303 K and 773 K.

Procedure for Rietveld refinements and Le Bail fittings: The Rietveld refinements and Le Bail fittings were performed by the program TOPAS 3.0 (Bruker AXS). The Pearson VII function and FP function were used for Rietveld refinements and Le Bail fittings, respectively. The contribution to the peak shape due to the experimental setup was obtained after performing a Rietveld refinement of the CeO_2 standard. The obtained parameters were kept fixed for the refinements on each data profile. The refinements by the structural models shown in Supplementary Tables provided the best fit to the XRD patterns.

Synthesis of fcc-Ru nanoparticles (NPs): To synthesize fcc-Ru NPs, a TEG solution (20 ml) containing $Ru(acac)_3$ (398.4 mg, 1 mmol) was added into a mixture solution of PVP (27.5 mg) and TEG (80 ml) at 240 ˚C under ambient condition. The reaction was kept for 5 min and cooled to room temperature. The dark brown solution was mixed with diethyl ether and acetone, and the mixture solution was centrifuged. The concentrate was washed with ethanol for several times to remove excess PVP amount and other byproducts. Finally, a black powder was collected by vacuum drying.

Synthesis of Cu NPs: To synthesize Cu NPs, a TEG solution (10 ml) containing $Cu(OAc)_2·H_2O$ (99.8 mg, 0.5 mmol) was added into a mixture solution of PVP (1.1 g) and TEG (100 ml) at 240 ˚C under N_2 bubbling. The reaction was kept for 10 min and cooled to room temperature. The black powder was collected by same post-treatment as fcc-Ru NPs.

X-ray photoelectron spectroscopy (XPS) measurements: XPS spectra for samples on a carbon sheet were analyzed using a Shimadzu ESCA-3400 X-ray photoelectron

spectrometer without surface etching treatment. The binding energies were corrected with reference to the C(1s) line at 284.5 eV. The Ru bulk and Cu bulk reagents for XPS measurements were purchased from Wako.

Catalyst preparation: The synthesized $Cu_{0.5}Ru_{0.5}$ and Ru NPs were supported on γ-Al_2O_3 catalysts by wet impregnation. Each NP (equivalent to 1 wt% of γ-Al_2O_3) was dispersed by ultrasonication in purified water. The γ-Al_2O_3 support has been precalcined at 1073 K for 5 h. The precalcined γ-Al_2O_3 was added into each NP solution, and then the suspended solutions were stirred for 12 h. After stirring, the suspended solutions were heated to 60 °C and dried under vacuum. The resulting powders were kept at 120 °C for 8 h for complete water removal. The loading amounts of metals were confirmed by XRF measurements. The obtained catalyst powders were pressed into pellets at 1.2 MPa for 5 min. The pellets were crushed and sieved to obtain grains with diameters between 180 and 250 μm.

Three-way catalysis (TWC) test: For the investigation of three-way catalytic activity, each catalyst (200 mg) was loaded into a tubular quartz reactor (internal diameter 7 mm) with quartz wool. A gas mixture of $NO/O_2/CO/C_3H_6/He$ ($NO/O_2/CO/C_3H_6$: 500/4050/5000/400 ppm) was passed over the catalysts at ambient temperature at space velocity at 60,000 $ml \cdot h^{-1} \cdot g^{-1}$. After 15 min, effluent gas was collected, and the reaction products were analyzed by gas chromatography with a thermal conductivity detector (GL SCIENCES 490-GC) and NO_x analyzer (Thermo Fisher Scientific, 42i-HL). The catalysts were heated in increments of 30 °C from 25 to 500 °C at 10°C/min, and the products were analyzed at each temperature. After the reaction, the reactor was purged with He at the reaction temperature, and the catalysts were then cooled to room temperature.

Definition of λ: The λ value of the simulated exhaust, which represents the ratio between the available oxygen and the oxygen needed for full conversion to CO_2, H_2O and N_2, is defined as $\lambda = \{2[O_2] + [NO]\}/\{9[C_3H_6] + [CO]\}$; λ = 1 at stoichiometry and the corresponding concentration of O_2 was 4050 ppm.

λ dependence test: For the investigation of λ dependence in three-way catalytic reaction, similar condition with TWC test was used. Temperature was fixed to 400 °C. O_2 amount was changed from 810 to 8100 ppm (calculated 4050 ppm as λ=1).

***In situ* Fourier transfer infrared (FTIR) measurements.** FTIR spectra of adsorbed CO and NO as a probe molecule were obtained to evaluate roles of Ru and Cu sites in NO_x reduction. A self-supporting sample disk of approximately 20 mg cm^{-2} placed in an IR cell with CaF_2 windows was first pre-treated at 100 °C in a flow of 5% H_2/He for 30 min and then purged with He at 200 °C for 10 min. The activated sample disk was first exposed to 1% CO/He gas flow at 200 °C for 20 min and then to 1% CO + 1% NO/He gas flow at 200 °C for 20 min. On the other hand, the reactivated sample disk was first exposed to 1% NO/He gas flow at 200 °C for 20 min and then to 1% CO + 1% NO/He gas flow at 200 °C for 20 min. All IR spectra were recorded at the interval of 30 seconds using a Nicolet Nexus 670 FTIR spectrometer, accumulating 64 scans at a resolution of 4 cm^{-1}.

Mass transfer calculations.

Thiele theory has been used to evaluate the influence of mass transfer factor.

In the NO reduction reaction (NO + CO → $1/2N_2$ + CO_2), the apparent reaction rate constant k_{NO} can be obtained from

$$k_{NO} = \frac{r_{NO}}{\theta_{CO}\theta_{NO}} \qquad (1)$$

At the condition of T = 673 K, P = 101 kPa, C_{NO} = 500 ppm, $GHSV$ = 12,000 h^{-1} (calculated from $WHSV$ = 60,000 ml $gcat^{-1}$ h^{-1}), NO conversion = 100%, apparent reaction rate of NO reduction is estimate as

$$r_{NO} = -\frac{dn_{NO}}{dt} \approx \frac{d\frac{PV_{NO}}{RT}}{dt} = \frac{P}{RT}\frac{dV_{NO}}{dt} = \frac{P}{RT}GHSV_{NO} \times conv._{NO} \approx 0.1\mu mol\ h^{-1}$$

Based on equation (1), if set θ_{CO} = 0.5, θ_{NO} = 0.1, the apparent reaction rate constant is calculated as k_{NO} = 0.002 mmol h^{-1}.

The Thiele modulus is:

$$\Phi_s = R_0\sqrt{\frac{k_{NO}C_{NOs}}{D_e}} \qquad (2)$$

R_0 is the radii of catalyst particle (ca. 100 nm), C_{NOs} is the NO concentration at particle surface, D_e is the effective diffusion coefficient.

When pore size is relative large, D_e can be calculated as

$$D_e = D\frac{\theta}{\tau} = \frac{1}{3}\bar{v}\lambda\frac{\theta}{\tau} \qquad (3)$$

$\bar{v}$ is the mean velocity of gas molecule, λ is mean free path, θ is porosity and τ is bending factor of pore.

From Maxwell's velocity distribution law,

$$\overline{v_{NO}} = \sqrt{\frac{8RT}{\pi M_{NO}}} = 689\ m/s$$

From Kinetic theory of gases,

$$\lambda_{NO} = \frac{k_B T}{\sqrt{2}\pi d_{NO}^2 p_T} = 52\ nm$$

Thus, from equation (3), if set θ = 0.5 (0.3~0.7 in most case) and τ = 3 (2~7 in most case), D_e=2.0×10^{-6} m^2 s^{-1}

Finally, from equation (2), we obtain Thiele modulus as Φs = 1.3×10^{-7}. The corresponding effectiveness factor is then estimated as $\boldsymbol{\eta}$ = 1 based on the $\boldsymbol{\Phi}$-$\boldsymbol{\eta}$ relation. Since Thiele modulus for NO reduction is extremely small, we can neglect the influence of mass transfer.

Heat transfer calculations.

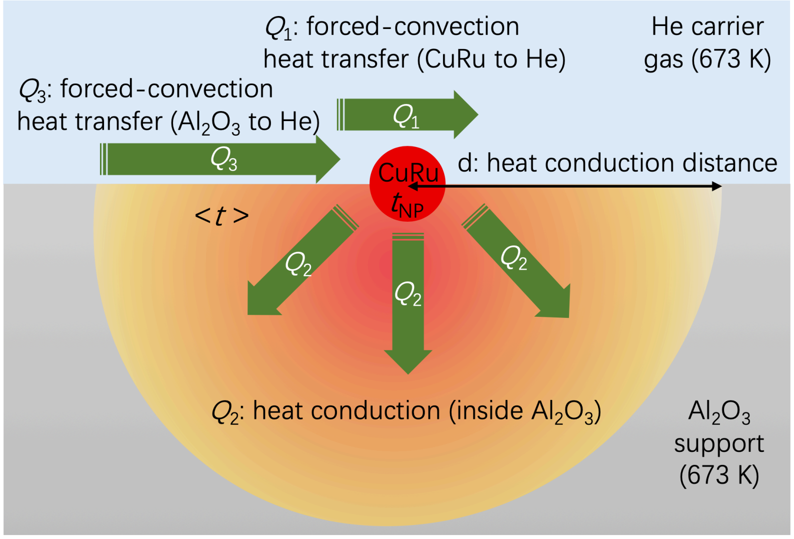

In order to simplify the heat transfer, we build up a model in the figure above. Three dependent processes are considered,

Q_1: forced-convection heat transfer (CuRu to He), $Q_1 = q_1A_1$;

Q_2: heat conduction (inside Al_2O_3), $Q_2 = q_2A_2$;

Q_3: forced-convection heat transfer (Al_2O_3 to He), $Q_3 = q_3A_3$.

Obviously, the reaction heat $Q_r = Q_1 + Q_2$.

Set d as the heat conduction distance in Al_2O_3, t_{NP} as the temperature of CuRu NP, $\langle t \rangle$ as the heat-convection equivalent average temperature of the A_3.

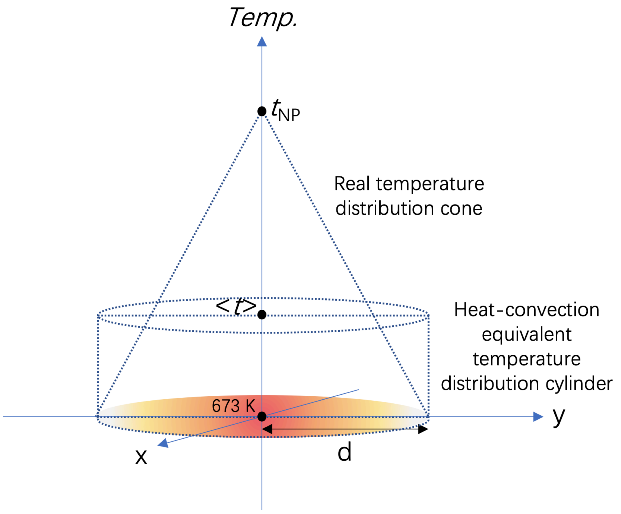

From the above figure, we can easily obtain $<t> = 673$ K + 1/3(t_{NP} − 673 K).

We set the forced-convection heat transfer coefficient of He in our experimental condition, h_{He} = 200 W m^{-2} K^{-1}.

Thus, $Q_3 = q_3A_3 = h_{He}(<t> - 673\ K)\ \pi d^2 = 209(t_{NP} - 673\ K)d^2\ W\ m^{-2}\ K^{-1}$ (1)

Thermal conductivity of Al_2O_3, λ_{Al2O3} = 30 W m^{-1} K^{-1}, the mean radius of CuRu NPs is set as 5 nm, thus the semi-sphere heat conduction process can be calculated as:

$$Q_2 = \frac{1}{2}\frac{t_{NP} - 673\ K}{\frac{1}{4\pi\lambda_{Al2O3}}\left(\frac{1}{5\times10^{-9}\ m} - \frac{1}{d}\right)} = 188\ W\ m^{-1}\ K^{-1}(t_{NP} - 673\ K)\frac{5\times10^{-9}\ m\cdot d}{d - 5\times10^{-9}\ m}$$

Since λ_{Al2O3} is very large, d≫$5\times10^{-9}\ m$,

$$\frac{d}{d - 5\times10^{-9}\ m} \approx 1$$

the above equation becomes

$$Q_2 = 9.4\times10^{-7}(t_{NP} - 673\ K)\ W\ K^{-1} \quad (2)$$

Because $Q_2 = Q_3$, combine equation (1) and (2), we can obtain heat conduction distance,

$$d = 9.5\times10^{-5}\ m$$

From standard formation enthalpies of reactants and products, if the entropy changes are neglected, we can easily obtain reaction enthalpy differences as follows,

NO + CO → 1/2N_2 + CO_2 $\Delta H_{r,NO\ red} = -373.3\ kJ\ mol^{-1}$

$CO + 1/2O_2 \rightarrow CO_2$ $\Delta H_{r,CO\ ox} = -565.9\ kJ\ mol^{-1}$

$C_3H_6 + 9/2O_2 \rightarrow 3CO_2 + 3H_2O$ $\Delta H_{r,HC\ ox} = -1926.5\ kJ\ mol^{-1}$

From $WHSV$ = 60,000 ml gcat^{-1} h^{-1}, the total flow rate ξ_{Tot} = 3.3 ml/s.

The reactant concentration are $NO/O_2/CO/C_3H_6$: 500/4050/5000/400 ppm, from $PV=nRT$, we calculate mole flow rate of every NO, CO and C_3H_6 as follows,

$$\xi_{NO} = 3.0\times10^{-13}\ \text{mol/s}$$

$$\xi_{CO} = 3.0\times10^{-12}\ \text{mol/s}$$

$$\xi_{C3H6} = 2.4\times10^{-13}\ \text{mol/s}$$

Thus, the total reaction heat power of the TWC reaction

$$Q_r = -\Delta H_{r,NO\ red}\xi_{NO} - \Delta H_{r,CO\ ox}(\xi_{CO} - \xi_{NO}) - \Delta H_{r,HC\ ox}\xi_{C3H6} = 2.1\times10^{-6}\ W \quad (3)$$

The mass of CuRu is 2 mg, density of CuRu is 10.7 g/cm^3, V_{CuRu} = 1.9×10^{17} nm^3

The mean radium of CuRu NP is 5 nm, so the single NP volume V_{NP} = 523 nm^3

Thus, the amount of nanoparticles for 2 mg of CuRu NPs

$N_{NP} = V_{CuRu}/V_{NP} = 3.7\times10^{14}$

In the case of the forced-convection heat transfer (CuRu to He), the convection area of each particle is the area of semi-sphere, thus $A_1 = N_{NP}A_{Semi} = 5.8\times10^{-2}$ m^2

Thus the Q_1 is calculated as follows,

$$Q_1 = q_1A_1 = h_{He}(t_{NP} - 673K)A_1 = 11.6(t_{NP} - 673K)\ W\ K^{-1} \quad (4)$$

Combine equation (2), (3) and (4), since $Q_1 \gg Q_2$, $Q_r = Q_1 + Q_2 \approx Q_1$

we obtain $t_{NP} = (673 + 1.8\times10^{-7})$ K (5)

in other words, CuRu NP is only 1.8×10^{-7} K hotter than the surroundings, a "hot spot" is excluded. Thus, the heat transfer influence to the TWC can be neglected.

We can also calculate Q_1, Q_2 and Q_3,

$$Q_1 = 2.1\times10^{-6}\ W$$

$$Q_2 = Q_3 = 1.7\times10^{-13}\ W$$

2. Details of reaction time measurements

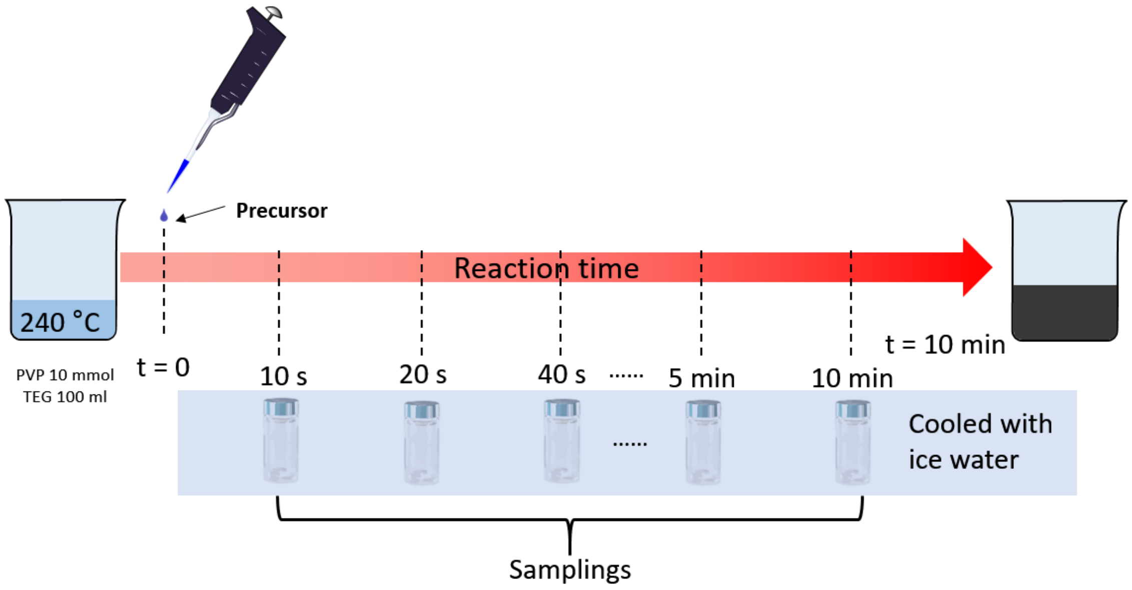

Fig. S1 General procedures of reaction time measurements.

Reaction time measurement of Ru acetylacetonate (Ru(acac)$_3$): 100 ml triethylene glycol (TEG) solution containing 1.1g polyvinylpyrrolidone (PVP) was heated to 240 ˚C under ambient condition (named as solution A). A precursor solution with 199.2 mg (0.5 mmol) Ru(acac)$_3$ dissolved in 10 ml TEG was dropwise added into solution A, the reaction system was stirred for 10 min under 240 ˚C. Samplings were taken at different time points as 1 min, 2 min, 3 min, 5 min and 8 min, respectively, followed by icy bath to cool the samples immediately. Transmission electron microscopy (TEM) measurements were carried out for each sample, reaction time was estimated by particle size change shown in Fig. 2a.

Reaction time measurement of Cu acetylacetonate (Cu(acac)$_2$): 100 ml TEG solution containing 1.1g PVP was heated to 240 ˚C under ambient condition (named as solution A). A precursor solution with 130.9 mg (0.5 mmol) Cu(acac)$_2$ dissolved in 10 ml TEG was dropwise added into solution A, the reaction system was stirred for 3 min under 240 ˚C. Samplings were taken at different time points as 10 s, 20 s, 40 s, 60 s and 90 s, respectively, followed by icy bath to cool the samples immediately. TEM measurements were carried out for each sample, reaction time was estimated by particle size change shown in Fig. S2.

Reaction time measurement of Cu acetate monohydrate (Cu(OAc)$_2$·H$_2$O): 100 ml TEG solution containing 1.1g PVP was heated to 240 ˚C under ambient condition (named as solution A). A precursor solution with 99.8 mg (0.5 mmol) Cu(OAc)$_2$·H$_2$O dissolved in 10 ml TEG was dropwise added into solution A, the reaction system was stirred for 10 min under 240 ˚C. Samplings were taken at different time points as 10 s, 20 s, 40 s, 1 min, 1.5 min, 2 min, 3min, 5min and 10 min, respectively, followed by icy bath to cool the samples immediately. Reaction time was estimated by colorimetry shown in Fig. 2b.

Reaction time measurement of Cu formate ($Cu(HCOO)_2{\cdot}4H_2O$): 100 ml TEG solution containing 1.1g PVP was heated to 240 ˚C under ambient condition (named as solution A). A precursor solution with 76.8 mg (0.5 mmol) $Cu(HCOO)_2{\cdot}4H_2O$ dissolved in 10 ml TEG was dropwise added into solution A, the reaction system was stirred for 10 min under 240 ˚C. Samplings were taken at different time points as 10 s, 20 s, 40 s, 1 min, 1.5 min, 2 min, 3min, 5min and 10 min, respectively, followed by icy bath to cool the samples immediately. Reaction time was estimated by colorimetry shown in Fig. 2c.

3. Reaction time measurement results and spatial structure of $Cu(acac)_2$

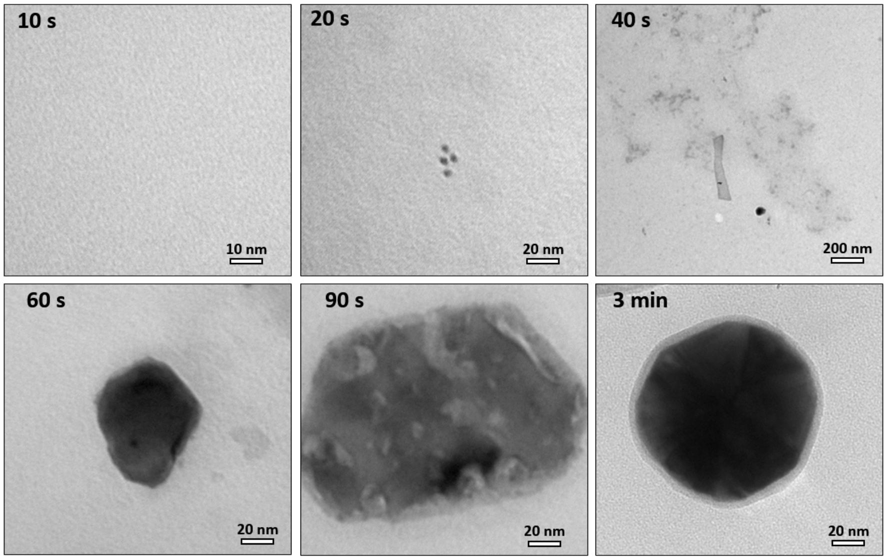

Fig. S2 Reaction time measurement results of $Cu(acac)_2$ by TEM measurement.

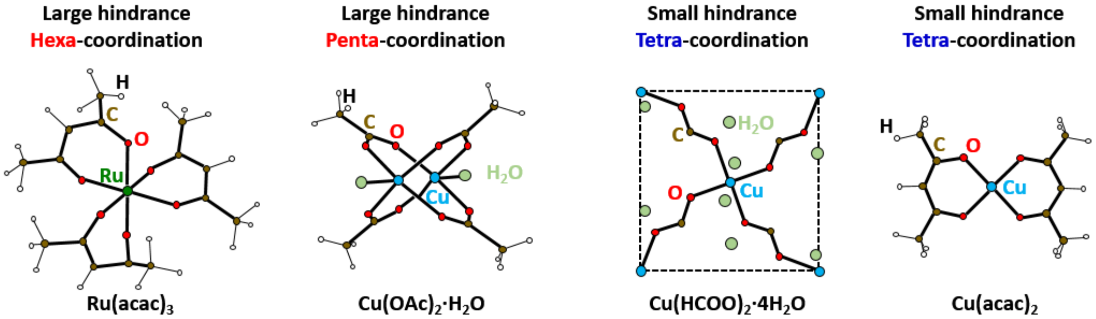

Fig. S3 Spatial structures of $Ru(acac)_3$, $Cu(OAc)_2{\cdot}H_2O$ dimer, $Cu(HCOO)_2{\cdot}4H_2O$ and $Cu(acac)_2$.

4. Reduction potentials of Cu and Ru

Table S1 Reduction potentials of Cu and Ru under standard conditions (25 °C, 1 atm). SHE: Standard Hydrogen Electrode[1]

Reduction Reaction	E^0 (V vs SHE)
$Cu^{2+} + 2e^- \rightarrow Cu$	0.3419
$Ru^{3+} + e^- \rightarrow Ru^{2+}$	0.2487
$Ru^{2+} + 2e^- \rightarrow Ru$	0.455

5. CuRu alloy synthesis by combination of $Ru(acac)_3$ and $Cu(acac)_2$

Synthetic procedure: To synthesize CuRu alloy NPs, a TEG solution (4 ml) containing $Ru(acac)_3$ (19.9 mg, 0.05 mmol) and $Cu(acac)_2$ (13.1 mg, 0.05 mmol) was added into a mixture solution of PVP (220 mg) and TEG (10 ml) at 240 ˚C under ambient condition. The reaction was kept for 10 min and cooled to room temperature. The black powder was collected by same post-treatment as fcc-Ru NPs.

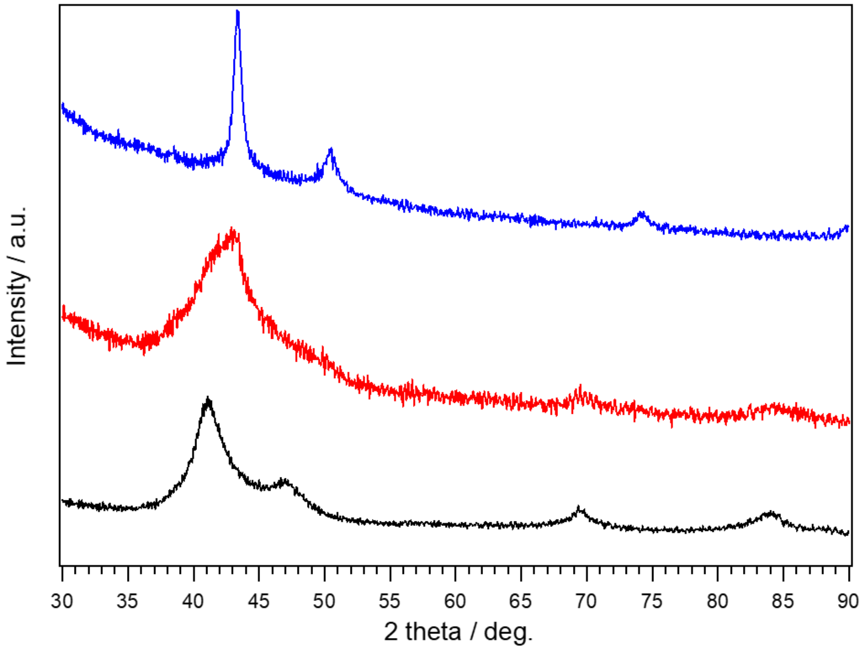

Fig. S4 XRD patterns of CuRu alloy NPs synthesized with $Ru(acac)_3$ and $Cu(acac)_2$ (red), compared with fcc-Ru NPs (black) and Cu NPs (blue) at 303 K. The radiation wavelength was 1.54056 Å.

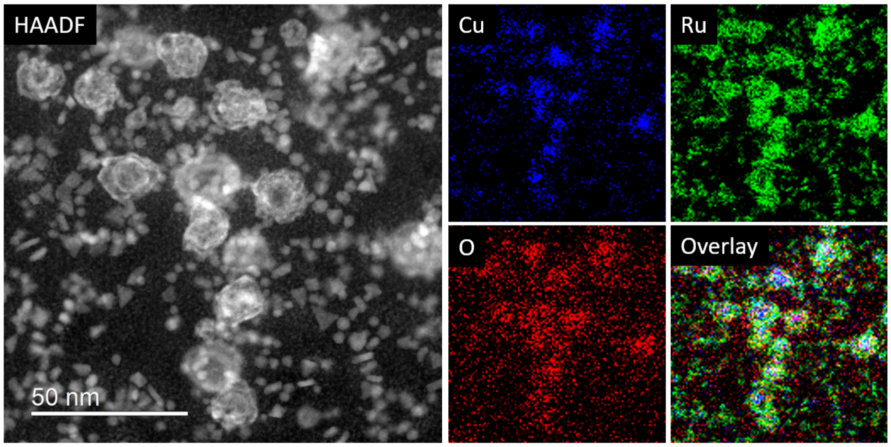

Fig. S5 High-angle annular dark-field scanning transmission electron microscopy (HAADF-STEM) image, Cu-K, Ru-L, O-K and reconstructed overlay STEM-EDX maps obtained from a group of CuRu alloy NPs synthesized from $Ru(acac)_3$ and $Cu(acac)_2$.

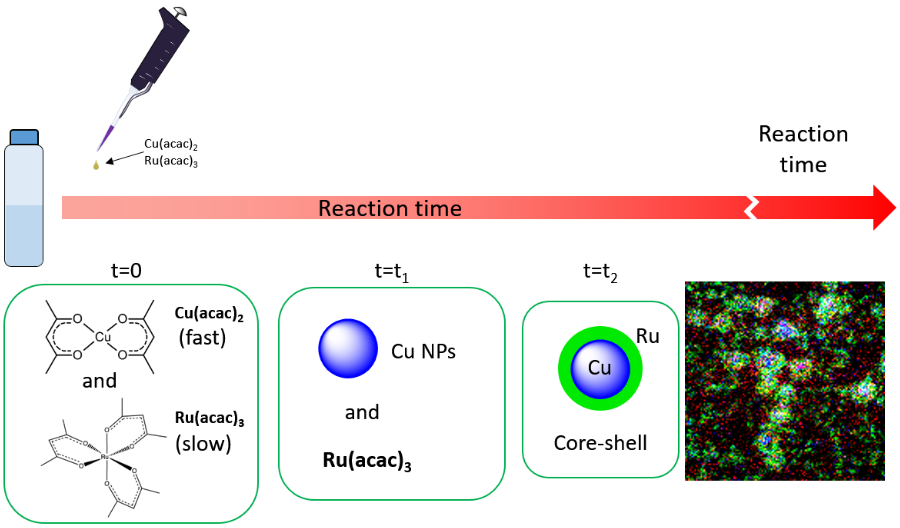

Fig. S6 Schematic illusion for Cu@Ru core-shell structure formation. According to core-shell formation mechanism, reaction time of $Cu(acac)_2$ was estimated to be much shorter than that of $Ru(acac)_3$, which is consistent with reaction time measurements of $Cu(acac)_2$ and $Ru(acac)_3$.

6. CuRu alloy synthesis by combination of $Ru(acac)_3$ and $Cu(HCOO)_2·4H_2O$

Synthetic procedure: To synthesize CuRu alloy NPs, a TEG solution (10 ml) containing $Ru(acac)_3$ (199.2 mg, 0.5 mmol) and $Cu(HCOO)_2·4H_2O$ (112.8 mg, 0.5 mmol) was added into a mixture solution of PVP (1.1 g) and TEG (100 ml) at 240 ˚C under ambient condition. The reaction was kept for 10 min and cooled to room temperature. The black powder was collected by same post-treatment as fcc-Ru NPs.

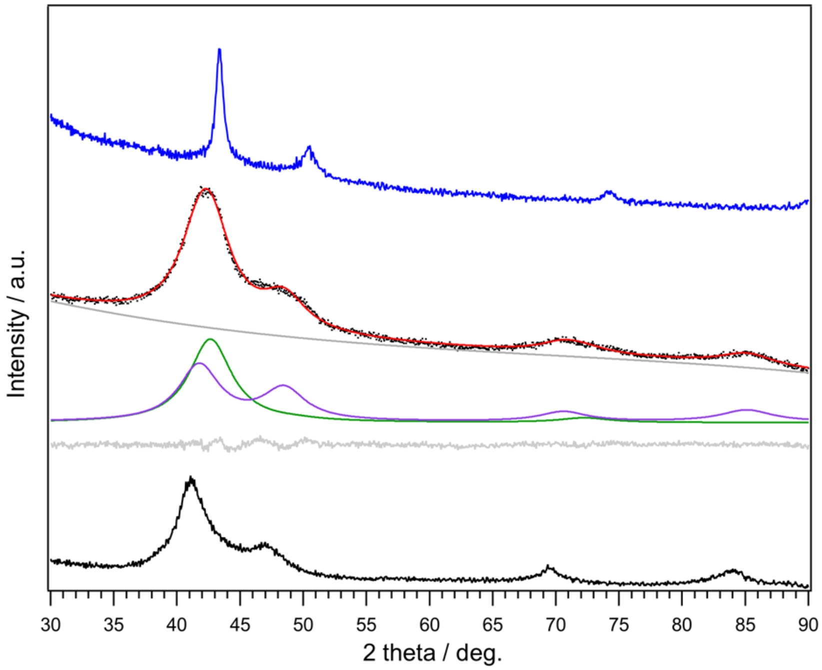

Fig. S7 XRD patterns of CuRu alloy NPs synthesized with Ru(acac)$_3$ and Cu(HCOO)$_2$·4H$_2$O (black dots), calculated pattern (red line), background (gray, upper), the fitting curves of the Ru rich component (purple) and Cu rich component (green), the difference profile (gray, bottom) from Le Bail fitting compared with fcc-Ru NPs (black) and Cu NPs (blue) at 303 K. The radiation wavelength was 1.54056 Å.

Table S2 Le Bail fitting results for CuRu alloy NPs synthesized with Ru(acac)$_3$ and Cu(HCOO)$_2$·4H$_2$O

T = 303 K, R_{wp} = 1.69%, GOF = 1.24

(a) Ru rich component, space group: *Fm*-3*m*

Lattice constant *a*	3.805(4) Å
Crystal size	2.4 nm

(b) Cu rich component, space group: *Fm*-3*m*

Lattice constant *a*	3.727(6) Å
Crystal size	2.3 nm

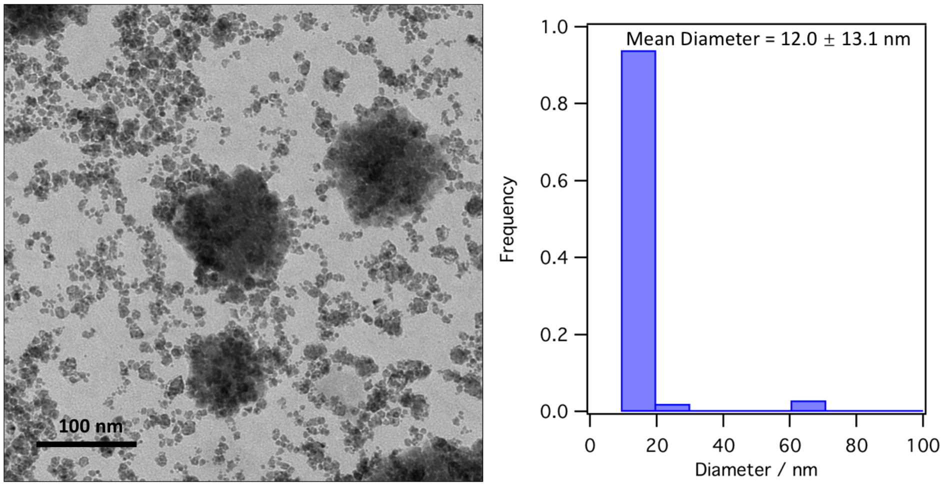

Fig. S8 TEM image and histogram of CuRu alloy NPs synthesized with $Ru(acac)_3$ and $Cu(HCOO)_2{\cdot}4H_2O$. Multiple components are shown.

7. CuRu alloy synthesis by combination of $Ru(acac)_3$ and $Cu(OAc)_2{\cdot}H_2O$

Synthetic procedure: To synthesize CuRu alloy NPs, a TEG solution (10 ml) containing $Ru(acac)_3$ (199.2 mg, 0.5 mmol) and $Cu(OAc)_2{\cdot}H_2O$ (99.8 mg, 0.5 mmol) was added into a mixture solution of PVP (1.1 g) and TEG (100 ml) at 240 ˚C under ambient condition. The reaction was kept for 10 min and cooled to room temperature. The black powder was collected by same post-treatment as fcc-Ru NPs.

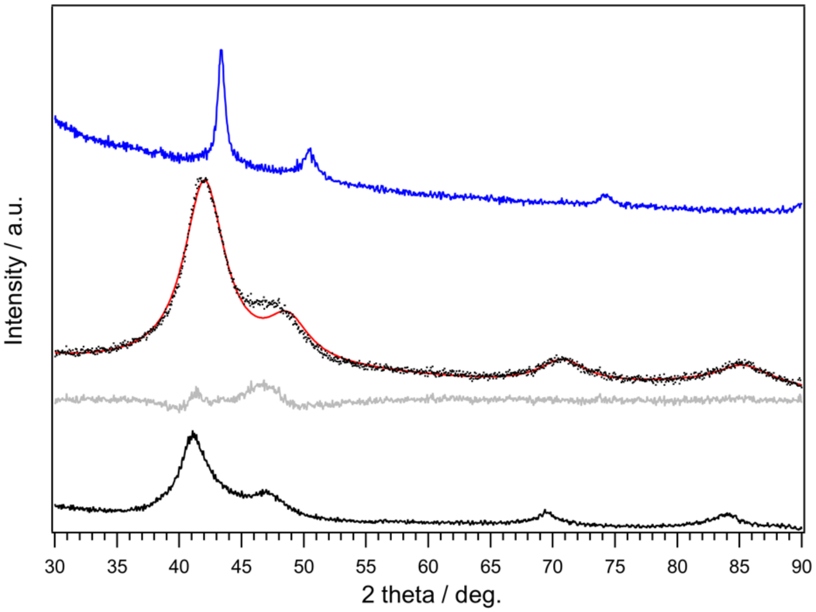

Fig. S9 XRD patterns of CuRu alloy NPs synthesized with $Ru(acac)_3$ and $Cu(OAc)_2{\cdot}H_2O$ (black dots), calculated pattern (red line), the difference profile (gray) from Le Bail fitting compared with fcc-Ru NPs (black) and Cu NPs (blue) at 303 K. The radiation wavelength was 1.54056 Å.

Table S3 Le Bail fitting results for CuRu alloy NPs synthesized with $Ru(acac)_3$ and $Cu(OAc)_2{\cdot}H_2O$

T = 303 K, R_{wp} = 3.04%, GOF = 2.14

space group: *Fm*-3*m*

Lattice constant *a*	3.775(1) Å
Crystal size	2.3 nm

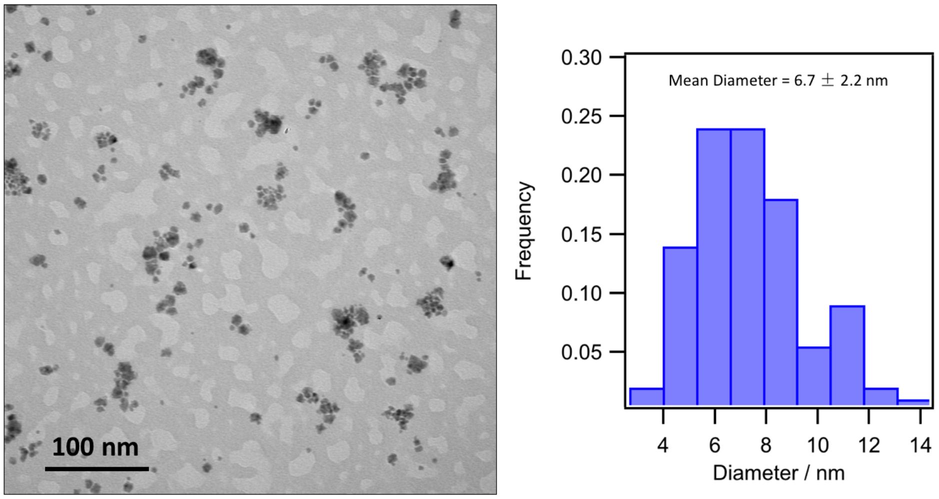

Fig. S10 TEM image and histogram of CuRu alloy NPs synthesized with $Ru(acac)_3$ and $Cu(OAc)_2{\cdot}H_2O$. Single distribution is shown.

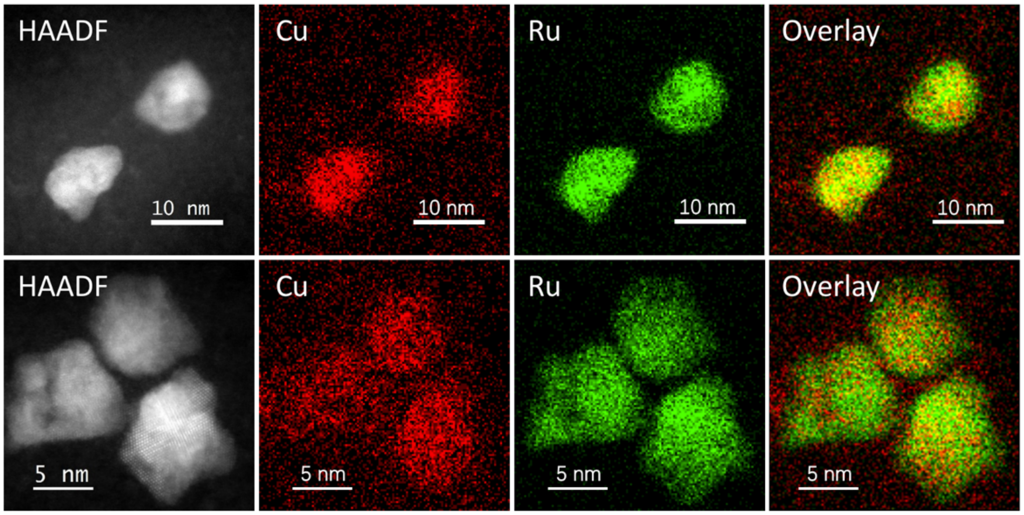

Fig. S11 HAADF-STEM image, Cu-K, Ru-L and reconstructed overlay STEM-EDX maps obtained from two group of CuRu alloy NPs synthesized from $Ru(acac)_3$ and $Cu(OAc)_2{\cdot}H_2O$.

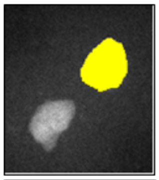

Element	Net	Net	K-Factor	Wt.%	Wt.%	Atom %	Atom %
Line	Counts	Error			Error		Error
Cu K	1845	±100	1.529	17.64	±0.96	**25.41**	± 1.38
Ru L	7219	±227	1.825	82.36	±2.59	**74.59**	± 2.35

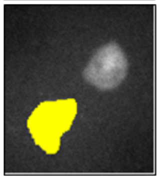

Element	Net	Net	K-Factor	Wt.%	Wt.%	Atom %	Atom %
Line	Counts	Error			Error		Error
Cu K	2586	±113	1.529	18.94	±0.83	**27.09**	± 1.18
Ru L	9273	±247	1.825	81.06	±2.16	**72.91**	± 1.94

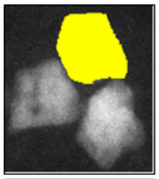

Element	Net	Net	K-Factor	Wt.%	Wt.%	Atom %	Error
Line	Counts	Error			Error		
Cu K	2584	±113	1.529	19.99	±0.88	**28.44**	± 1.24
Ru L	8664	±247	1.825	80.01	±2.28	**71.56**	± 2.04

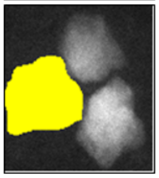

Element	Net	Net	K-Factor	Wt.%	Wt.%	Atom %	Error
Line	Counts	Error			Error		
Cu K	2894	±127	1.529	15.23	±0.67	**22.23**	± 0.98
Ru L	13495	±300	1.825	84.77	±1.88	**77.77**	± 1.73

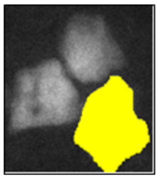

Element	Net	Net	K-Factor	Wt.%	Wt.%	Atom %	Atom %
Line	Counts	Error			Error		Error
Cu K	3600	±138	1.529	16.22	±0.62	**23.55**	± 0.90
Ru L	15576	±319	1.825	83.78	±1.72	**76.45**	± 1.57

Fig. S12 Select area analyses of each particle in STEM-EDX mapping of CuRu alloy NPs synthesized from Ru(acac)$_3$ and Cu(OAc)$_2$·H_2O. The atomic ratio of Cu:Ru was nearly equal to 1:3, which is far from the nominal ratio 1:1. The difference may due to Cu oxidation during synthetic procedure.

8. CuRu alloy synthesis by combination of $Ru(acac)_3$ and $Cu(OAc)_2{\cdot}H_2O$ with anhydrous solvent

Synthetic procedure: The procedure was the same with CuRu alloy synthesis by combination of $Ru(acac)_3$ and $Cu(OAc)_2{\cdot}H_2O$ except for solvent pretreatment. Solvent TEG in this synthesis was pretreated with activated molecular sieves overnight to remove water, and freshly treated anhydrous TEG was soon used in synthesis after pretreatment.

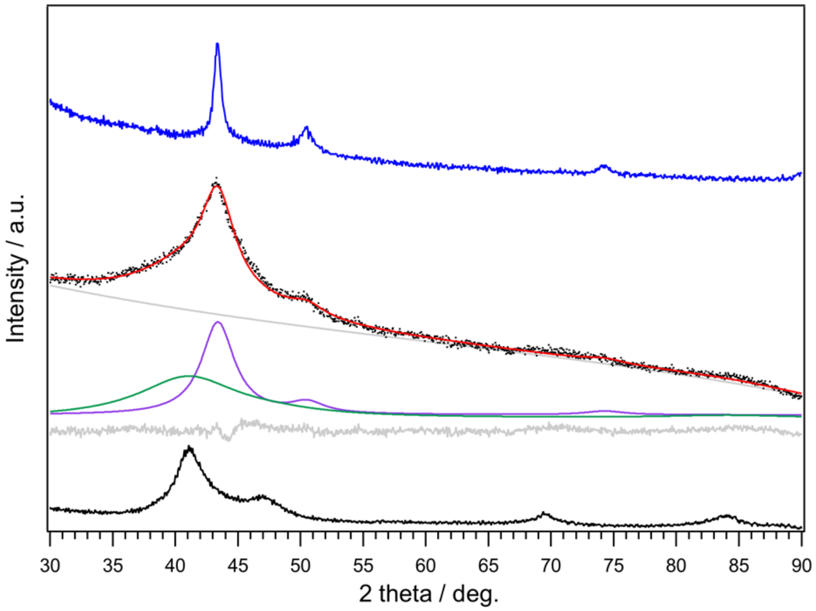

Fig. S13 XRD patterns of CuRu alloy NPs synthesized with $Ru(acac)_3$, $Cu(OAc)_2{\cdot}H_2O$ and anhydrous solvent (black dots), calculated pattern (red line), background (gray, upper), the fitting curves of the Ru rich component (green) and Cu rich component (purple), the difference profile (gray, bottom) from Le Bail fitting compared with fcc-Ru NPs (black) and Cu NPs (blue) at 303 K. The radiation wavelength was 1.54056 Å.

Table S4 Le Bail fitting results for CuRu alloy NPs synthesized with $Ru(acac)_3$ and $Cu(OAc)_2{\cdot}H_2O$ and anhydrous solvent

T = 303 K, R_{wp} = 1.51%, GOF = 1.31

(a) fcc-Ru component, space group: *Fm*-3*m*

Lattice constant *a*	3.81(2) Å
Crystal size	1.0 nm

(b) Cu component, space group: *Fm*-3*m*

Lattice constant *a*	3.612(1) Å
Crystal size	3.0 nm

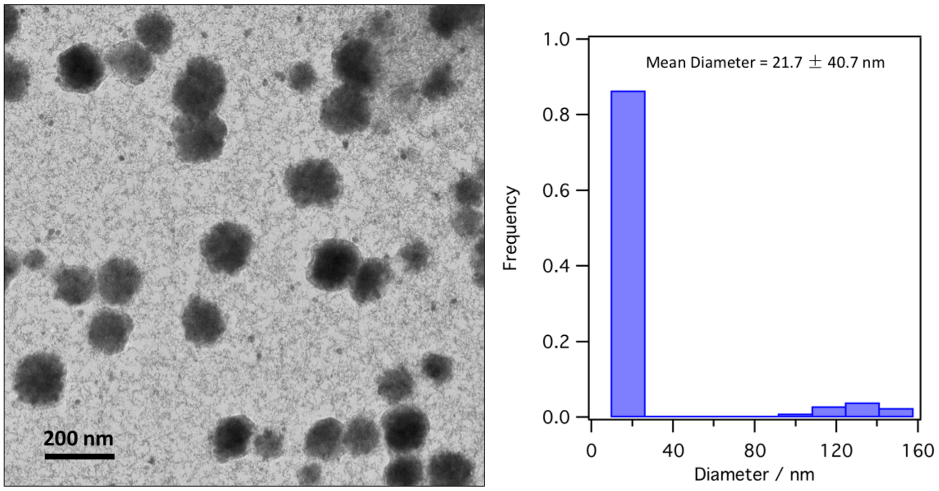

Fig. S14 TEM image and histogram of CuRu alloy NPs synthesized with $Ru(acac)_3$ and $Cu(OAc)_2{\cdot}H_2O$ and anhydrous solvent. Multiple components are shown.

9. Temperature factor optimization.

Synthetic procedure: The procedure was the same with CuRu alloy synthesis by combination of $Ru(acac)_3$ and $Cu(OAc)_2{\cdot}H_2O$ except for higher temperature of 270 ˚C. Multiple components from PXRD were observed.

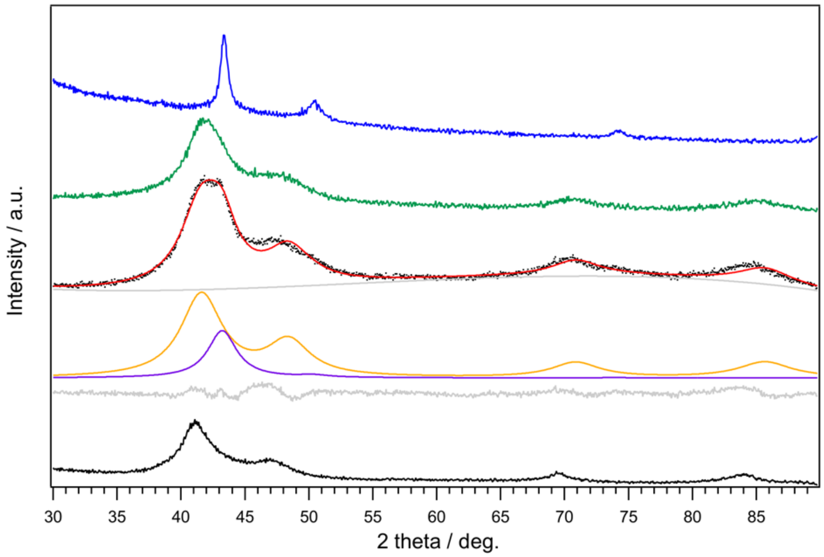

Fig. S15 XRD patterns of CuRu alloy NPs synthesized with $Ru(acac)_3$ and $Cu(OAc)_2{\cdot}H_2O$ at 240 (green, same pattern in Fig. S9) and 270 ˚C (black dots), calculated pattern (red line), background (gray, upper), the fitting curves of the Ru rich component (orange) and Cu rich component (purple), the difference profile (gray, bottom) from Le Bail fitting compared with fcc-Ru NPs (black) and Cu NPs (blue) at 303 K. The radiation wavelength was 1.54056 Å.

Table S5 Le Bail fitting results for CuRu alloy NPs synthesized with $Ru(acac)_3$ and $Cu(OAc)_2 \cdot H_2O$ at 270 ˚C

T = 303 K, R_{wp} = 2.41%, GOF = 1.78

(a) Ru rich component, space group: *Fm*-3*m*

Lattice constant *a*	3.761(3) Å
Crystal size	2.4 nm

(b) Cu rich component, space group: *Fm*-3*m*

Lattice constant *a*	3.626(2) Å
Crystal size	3.5 nm

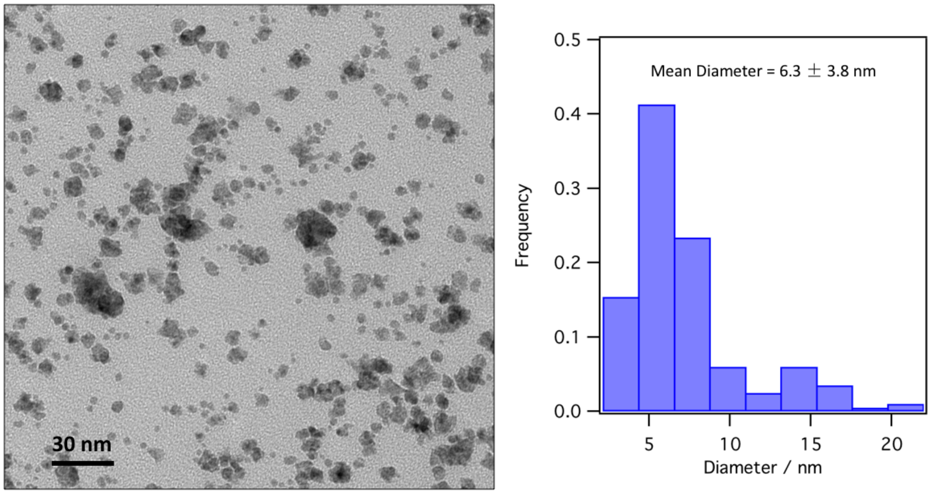

Fig. S16 TEM image and histogram of CuRu alloy NPs synthesized with $Ru(acac)_3$ and $Cu(OAc)_2 \cdot H_2O$ at 270 ˚C. Multiple components are shown.

10. Atmosphere factor optimization.

Synthetic procedure: The procedure was the same with CuRu alloy synthesis by combination of Ru(acac)$_3$ and Cu(OAc)$_2$·H$_2$O except for N_2 bubbling condition compared with ambient condition. Before reaction, TEG solvent was pre-bubbled with N_2 for 1 h. The N_2 bubbling was continued during reaction and was stopped until the reaction cooled down to room temperature.

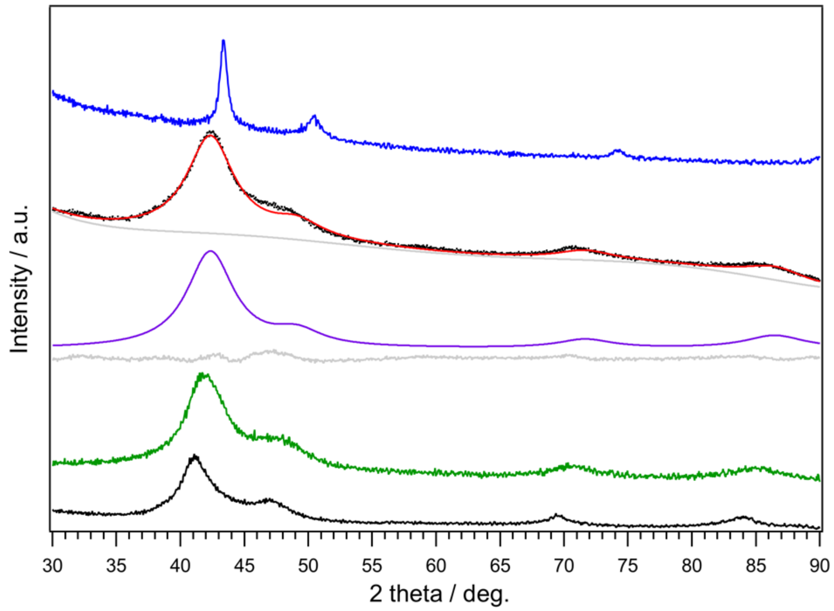

Fig. S17 XRD patterns of CuRu alloy NPs synthesized with Ru(acac)$_3$ and Cu(OAc)$_2$·H$_2$O at ambient condition (green, same pattern in Fig. S9) and N_2 bubbling condition (black dots), calculated pattern (red line), background (gray, upper), the fitting curves of fcc-alloy component (purple), the difference profile (gray, bottom) from Le Bail fitting compared with fcc-Ru NPs (black) and Cu NPs (blue) at 303 K. The radiation wavelength was 1.54056 Å.

Table S6 Le Bail fitting results for CuRu alloy NPs synthesized with Ru(acac)$_3$ and Cu(OAc)$_2$·H$_2$O at N_2 bubbling condition

T = 303 K, R_{wp} = 2.06%, GOF = 1.81

fcc-alloy component: space group: *Fm*-3*m*

Lattice constant *a*	3.757(3) Å
Crystal size	2.1 nm

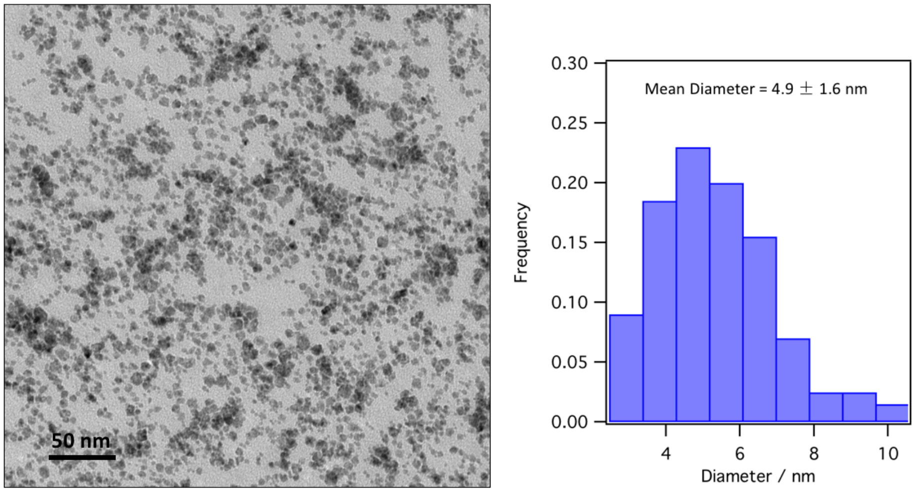

Fig. S18 TEM image and histogram of CuRu alloy NPs synthesized with $Ru(acac)_3$ and $Cu(OAc)_2{\cdot}H_2O$ at N_2 bubbling condition. Single distribution is shown.

Compared with the lattice constant of ambient condition sample (3.775 Å), the lattice constant of N_2 bubbling sample was decreased to 3.757(3) Å, which suggest a higher Cu content by N_2 bubbling. From this result, we could conclude that oxidation of Cu occurred during reaction. Those oxidized Cu cannot form alloy with Ru from the STEM-EDX mapping result in Figs. S11, 12.

11. Reducing agent and solvent factor optimization.

Synthetic procedure:

Diethylene glycol (DEG) solvent: Before reaction, a DEG solvent was pre-bubbled with N_2 for 1 h. To synthesize CuRu alloy NPs, a DEG solution (10 ml) containing $Ru(acac)_3$ (199.2 mg, 0.5 mmol) and $Cu(OAc)_2{\cdot}H_2O$ (99.8 mg, 0.5 mmol) was added into a mixture solution of PVP (1.1 g, 10 mmol) and DEG (100 ml) at 240 ˚C under N_2 bubbling. The N_2 bubbling was continued during reaction and was stopped until the reaction cooled down to room temperature. The reaction was kept for 10 min and cooled to room temperature. The black powder was collected by same post-treatment as fcc-Ru NPs.

Glycerol solvent: The procedure was the same with CuRu alloy synthesis procedure with DEG solvent above except for glycerol solvent.

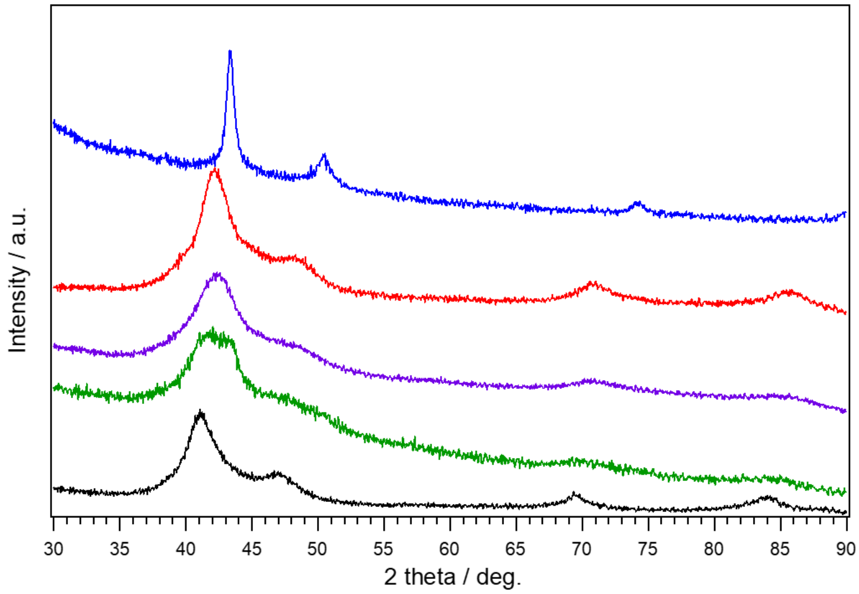

Fig. S19 XRD patterns of CuRu alloy NPs synthesized with $Cu(OAc)_2{\cdot}H_2O$ in diethylene glycol (DEG, red), TEG (purple, same pattern in Fig. S9) and glycerol (green), compared with fcc-Ru NPs (black) and Cu NPs (blue) at 303 K. The radiation wavelength was 1.54056 Å.

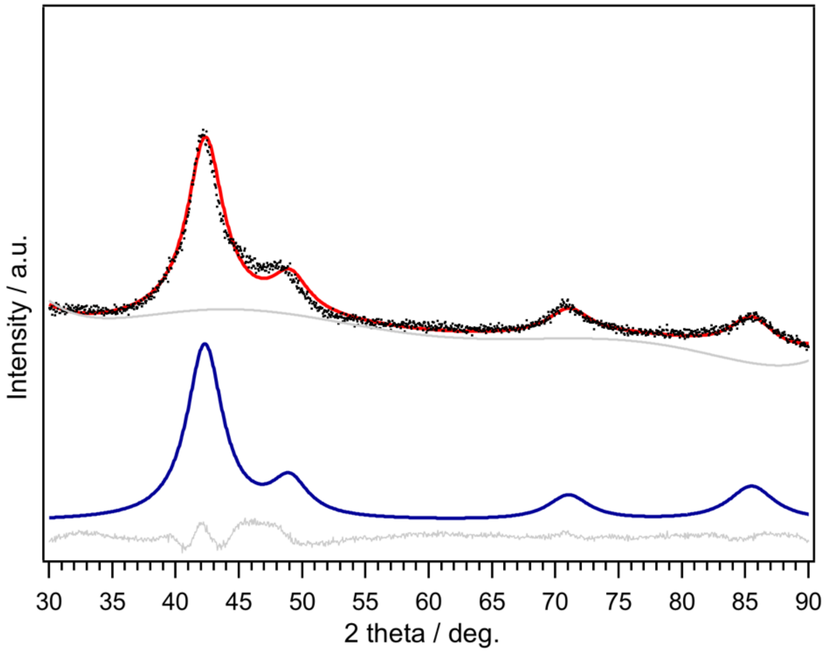

Fig. S20 XRD patterns of CuRu alloy NPs synthesized with $Ru(acac)_3$, $Cu(OAc)_2{\cdot}H_2O$ and DEG solvent (black dots), calculated pattern (red line), background (gray, upper), the fitting curves of the fcc component (blue) and the difference profile (gray, bottom) from Le Bail fitting at 303 K. The radiation wavelength was 1.54056 Å.

Table S7 Le Bail fitting results for CuRu alloy NPs synthesized with $Ru(acac)_3$, $Cu(OAc)_2{\cdot}H_2O$ and DEG solvent

T = 303 K, R_{wp} = 2.96%, GOF = 2.49

fcc-alloy component: space group: *Fm*-3*m*

Lattice constant *a*	3.758(7) Å
Crystal size	3.8 nm

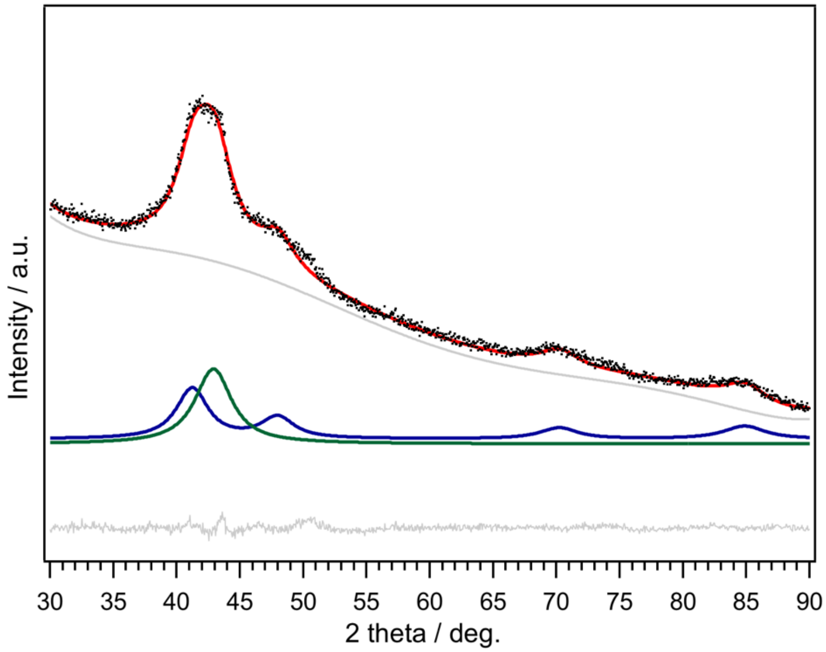

Fig. S21 XRD patterns of CuRu alloy NPs synthesized with $Ru(acac)_3$, $Cu(OAc)_2{\cdot}H_2O$ and glycerol solvent (black dots), calculated pattern (red line), background (gray, upper), the fitting curves of the Ru rich component (blue), Cu rich component (green) and the difference profile (gray, bottom) from Le Bail fitting at 303 K. The radiation wavelength was 1.54056 Å.

Table S8 Le Bail fitting results for CuRu alloy NPs synthesized with $Ru(acac)_3$, $Cu(OAc)_2{\cdot}H_2O$ and glycerol solvent

T = 303 K, R_{wp} = 1.77%, GOF = 1.29

(a) Ru rich component, space group: *Fm*-3*m*

Lattice constant *a*	3.792(5) Å
Crystal size	3.1(1) nm

(b) Cu rich component, space group: *Fm*-3*m*

Lattice constant *a*	3.639(5) Å
Crystal size	3.6(1) nm

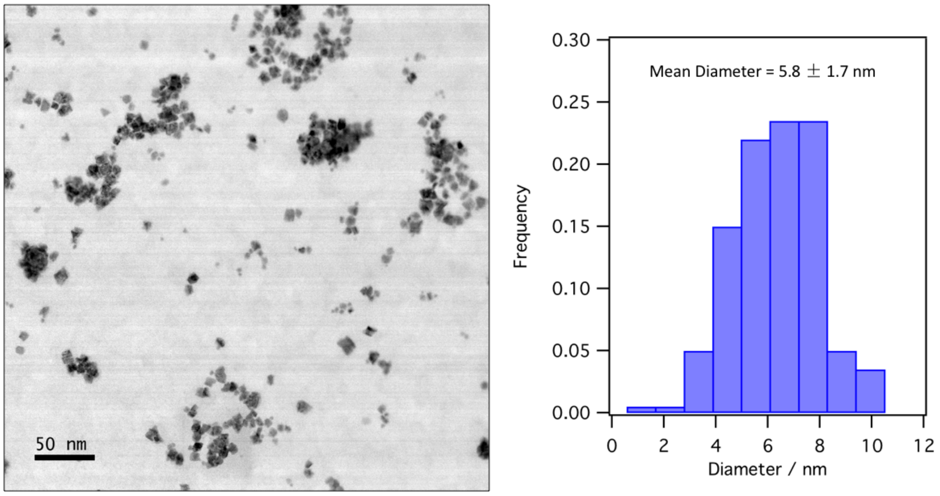

Fig. S22 STEM image and histogram of CuRu alloy NPs synthesized with $Ru(acac)_3$, $Cu(OAc)_2{\cdot}H_2O$ and DEG solvent at N_2 bubbling condition. Single distribution is shown.

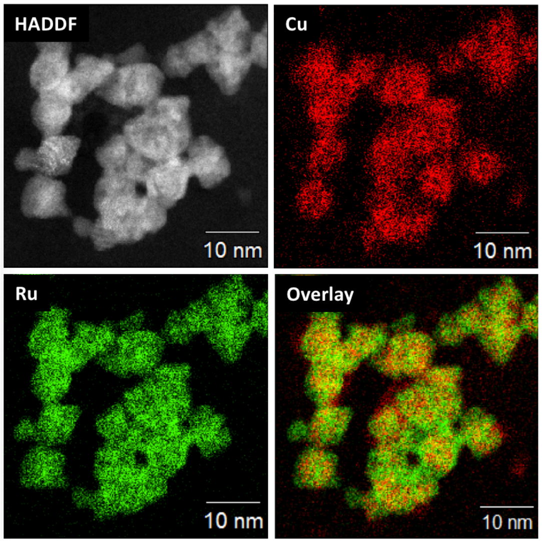

Fig. S23 HAADF-STEM image, Cu-K, Ru-L and reconstructed overlay STEM-EDX maps obtained from CuRu alloy NPs synthesized with $Ru(acac)_3$, $Cu(OAc)_2{\cdot}H_2O$ and DEG solvent at N_2 bubbling condition.

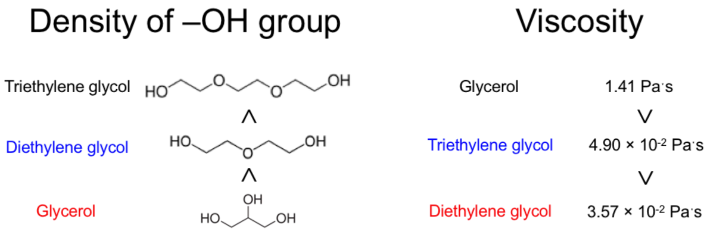

Scheme S1 XRD patterns of CuRu alloy NPs synthesized with $Cu(OAc)_2{\cdot}H_2O$ in diethylene glycol (DEG, red), TEG (purple) and glycerol (green), compared with fcc-Ru NPs (black) and Cu NPs (blue) at 303 K. The radiation wavelength was 1.54056 Å.

Product synthesized in DEG shows similar lattice constant compared with product obtained in TEG. On the other hand, multiple components product was obtained in glycerol (Fig. S21). In general, the reducing abilities of polyol are enhanced with the increasing of hydroxyl (-OH) group density. Stronger reducing ability provides faster reducing velocity. Thus, the reducing velocity ranking of the solvents is glycerol > DEG > TEG (Scheme S1). This velocity ranking did not fit with the quality ranking of nanoparticles synthesized in the solvents, which were DEG > TEG > glycerol. In this case, the qualities of different products are considered to highly correlate with the viscosities of solvents, which has also been reported by Park and coworkers in case of size control[2]. Considering that viscosity of glycerol (1.41 Pa·s) is nearly 2-order higher than those of DEG (3.57×10^{-2} Pa·s) and TEG (4.90×10^{-2} Pa·s) under room temperature (Scheme S1)[1], slower diffusion of metal atoms in glycerol would be expected, which resulted in inhomogeneous aggregation even with same reaction times. In contrast, DEG with lower viscosity can obtain the best quality of product among them.

12. Solvent oxygen content factor optimization.

Synthetic procedure: The procedure was the same with CuRu alloy synthesis by combination of $Ru(acac)_3$ and $Cu(OAc)_2{\cdot}H_2O$ except for 3-day-liquid-N_2 degassed DEG compared with N_2 bubbled DEG.

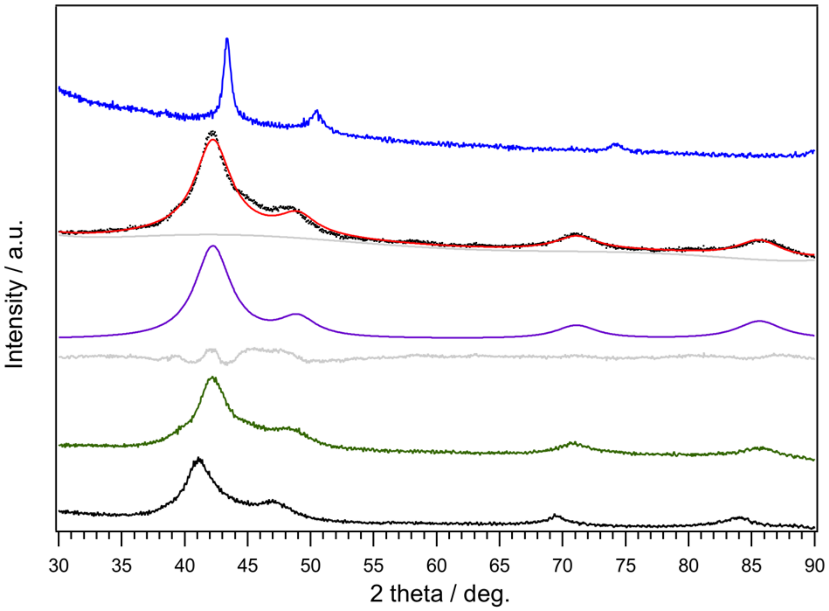

Fig. S24 XRD patterns of CuRu alloy NPs synthesized with $Ru(acac)_3$ and $Cu(OAc)_2{\cdot}H_2O$ in N_2 bubbled DEG (green, same pattern in Fig. S20) and liquid N_2 degassed DEG (black dots), calculated pattern (red line), background (gray, upper), the fitting curves of fcc-alloy component (purple), the difference profile (gray, bottom) from Le Bail fitting compared with fcc-Ru NPs (black) and Cu NPs (blue) at 303 K. The radiation wavelength was 1.54056 Å.

Table S9 Le Bail fitting results for CuRu alloy NPs synthesized with $Ru(acac)_3$, $Cu(OAc)_2{\cdot}H_2O$ and liquid N_2 degassed DEG

T = 303 K, R_{wp} = 2.89%, GOF = 2.46

fcc-alloy component: space group: *Fm*-3*m*

Lattice constant *a*	3.753(9) Å
Crystal size	2.8 nm

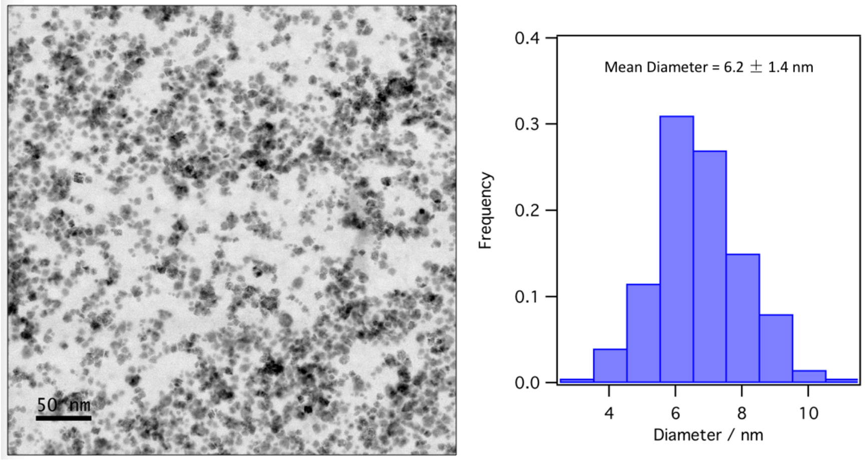

Fig. S25 STEM image and histogram of CuRu alloy NPs synthesized with $Ru(acac)_3$, $Cu(OAc)_2{\cdot}H_2O$ and DEG solvent at liquid N_2 degassing condition. Single distribution is shown.

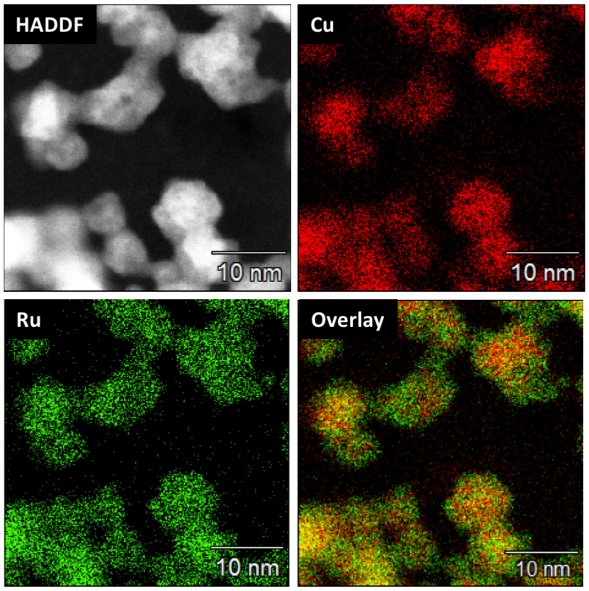

Fig. S26 HAADF-STEM image, Cu-K, Ru-L and reconstructed overlay STEM-EDX maps obtained from two group of CuRu alloy NPs synthesized from $Ru(acac)_3$ and $Cu(OAc)_2{\cdot}H_2O$ in liquid N_2 degassed DEG.

For degassed DEG sample, from the Le Bail fitting result of PXRD pattern, only slight decrease on lattice constant could be observed compared with N_2 bubbled DEG sample. Furthermore, by STEM-EDX mapping measurements, pure Cu elements locating on particle surface were found in N_2 bubbled DEG sample (Fig. S23), meanwhile most of Cu elements in liquid N_2 degassed DEG sample located inside CuRu alloy nanoparticles. This gives a direct evidence to better mixing for liquid N_2 degassed DEG than that of N_2 bubbled DEG. Solvent remaining oxygen by liquid N_2 degas could be fully removed, however N_2 bubbling cannot.

13. Stirring speed factor optimization.

Synthetic procedure: The reaction with $Ru(acac)_3$ and $Cu(OAc)_2·H_2O$ was carried out at stirring speed of 1200 rpm compared with 400 rpm under degassed condition. Very obvious right shift was observed for sample synthesized at stirring speed of 1200 rpm compared with that of stirring speed of 400 rpm. Better alloy formation was achieved at higher stirring speed. Combining the previous discussion for viscosity effect of solvents, it is clear that the diffusion process of metal atoms is of high importance in alloy formation for immiscible systems.

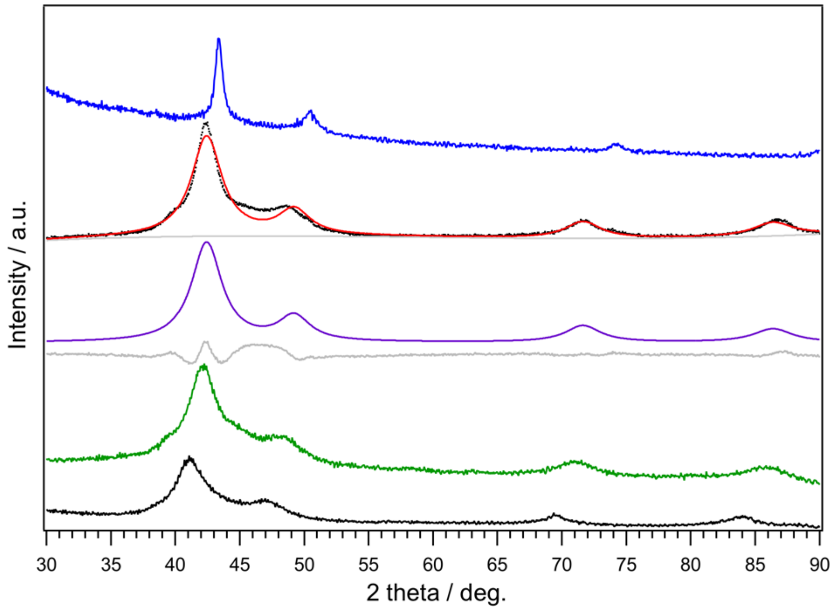

Fig. S27 XRD patterns of CuRu alloy NPs synthesized with $Ru(acac)_3$ and $Cu(OAc)_2·H_2O$ at the stirring speed of 400 rpm (green, same pattern in Fig. S24) and 1200 rpm (black dots), calculated pattern (red line), background (gray, upper), the fitting curves of fcc-alloy component (purple), the difference profile (gray, bottom) from Le Bail fitting compared with fcc-Ru NPs (black) and Cu NPs (blue) at 303 K. The radiation wavelength was 1.54056 Å.

Table S10 Le Bail fitting results for CuRu alloy NPs synthesized with $Ru(acac)_3$, $Cu(OAc)_2·H_2O$ at the stirring speed of 1200 rpm

T = 303 K, R_{wp} = 3.67%, GOF = 3.77

fcc-alloy component: space group: *Fm*-3*m*

Lattice constant *a*	3.725(3) Å
Crystal size	3.9 nm

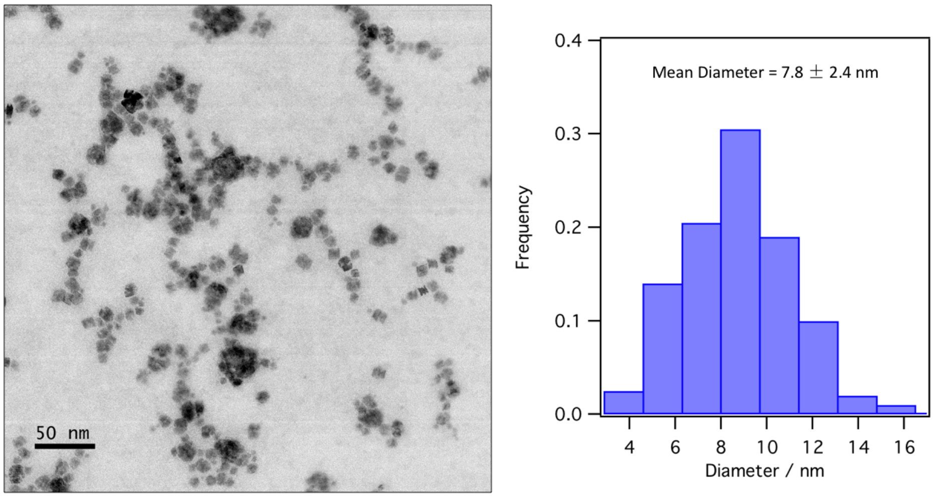

Fig. S28 STEM image and histogram of CuRu alloy NPs synthesized with Ru(acac)$_3$, Cu(OAc)$_2$·H$_2$O at stirring speed of 1200 rpm. Single distribution is shown.

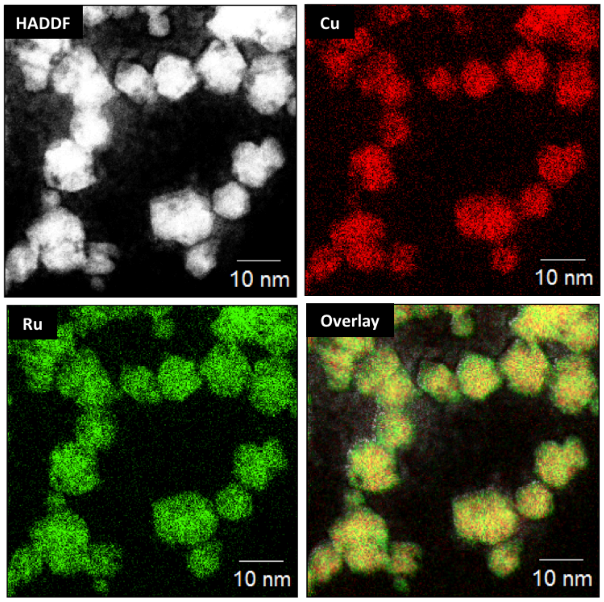

Fig. S29 HAADF-STEM image, Cu-K, Ru-L and reconstructed overlay STEM-EDX maps obtained from two group of CuRu alloy NPs synthesized from Ru(acac)$_3$ and Cu(OAc)$_2$·H$_2$O at stirring speed of 1200 rpm.

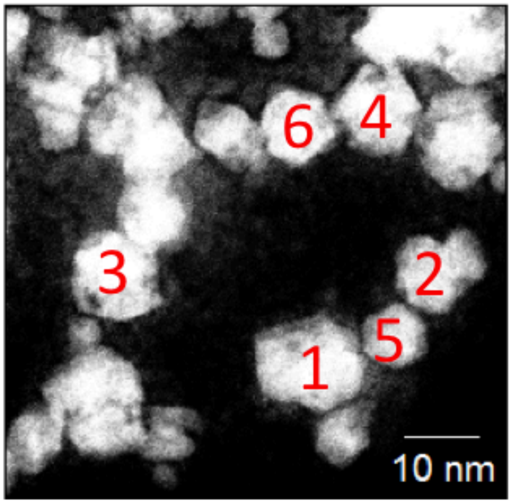

Whole area:							
Element Line	Net Counts	Net Error	K-Factor	Element Wt.%	Wt.% Error	Atom %	Atom % Error
Cu K	80774	±589	1.34	35.12	±0.26	46.27	± 0.34
Ru L	116645	±1073	1.713	64.88	±0.60	53.73	± 0.49
Total				100		100	

Area 1:							
Element Line	Net Counts	Net Error	K-Factor	Element Wt.%	Wt.% Error	Atom %	Atom % Error
Cu K	6837	±159	1.34	34.1	±0.79	45.14	± 1.05
Ru L	10330	±261	1.713	65.9	±1.66	54.86	± 1.39
Total				100		100	

Area 2:							
Element Line	Net Counts	Net Error	K-Factor	Element Wt.%	Wt.% Error	Atom %	Atom % Error
Cu K	3628	±116	1.34	30.65	±0.98	41.28	± 1.32
Ru L	6416	±200	1.713	69.35	±2.16	58.72	± 1.83
Total				100		100	

Area 3:							
Element Line	Net Counts	Net Error	K-Factor	Element Wt.%	Wt.% Error	Atom %	Atom % Error
Cu K	4737	±135	1.34	31.56	±0.90	42.31	± 1.21
Ru L	8032	±234	1.713	68.44	±1.99	57.69	± 1.68
Total				100		100	

Area 4:							
Element Line	Net Counts	Net Error	K-Factor	Element Wt.%	Wt.% Error	Atom %	Atom % Error
Cu K	4701	±131	1.34	33.07	±0.92	44	± 1.23
Ru L	7439	±218	1.713	66.93	±1.97	56	± 1.64
Total				100		100	

Area 5:							
Element Line	Net Counts	Net Error	K-Factor	Element Wt.%	Wt.% Error	Atom %	Atom % Error
Cu K	2271	±91	1.34	35.78	±1.44	46.98	± 1.88
Ru L	3187	±149	1.713	64.22	±3.01	53.02	± 2.48
Total				100		100	

Area 6:							
Element Line	Net Counts	Net Error	K-Factor	Element Wt.%	Wt.% Error	Atom %	Atom % Error
Cu K	3125	±107	1.34	30.01	±1.02	40.54	± 1.39
Ru L	5698	±190	1.713	69.99	±2.34	59.46	± 1.98
Total				100		100	

Fig. S30 Select area analyses of each particle in STEM-EDX mapping of CuRu alloy NPs synthesized from Ru(acac)$_3$ and Cu(OAc)$_2$·H_2O at stirring speed of 1200 rpm. The atomic ratio of Cu:Ru was nearly equal to 1:1 of the nominal ratio. Cu elements had been well mixed in.

14. XRF measurements

Table S11 Raw weight ratios and the corresponding atomic ratios for CuRu NPs determined by XRF measurements.

Sample Name	Ru wt%	Cu wt%	Ru at%	Cu at%
CuRu	64.8429	35.1571	53.7	46.3

15. HR-STEM measurements

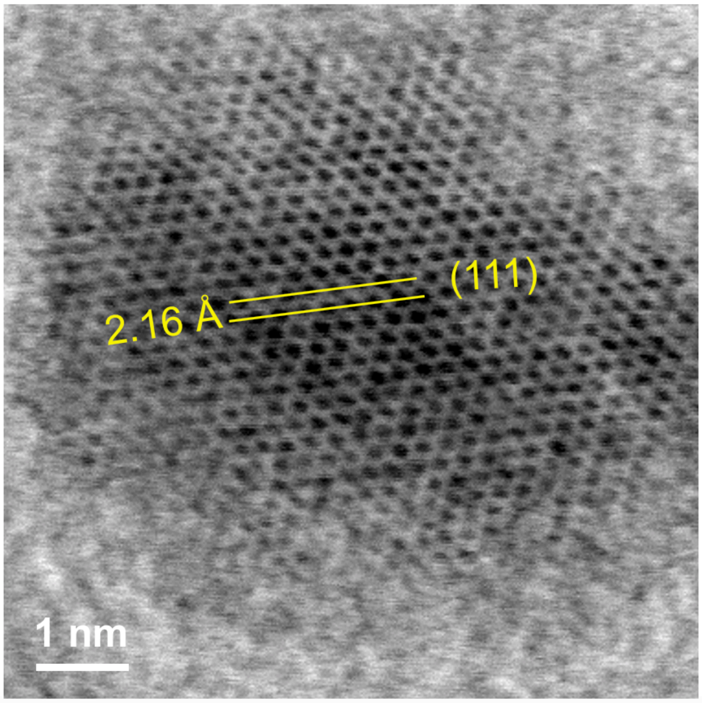

Fig. S31 Atomic resolution HR-STEM image of the CuRu nanoparticles.

16. XPS measurements

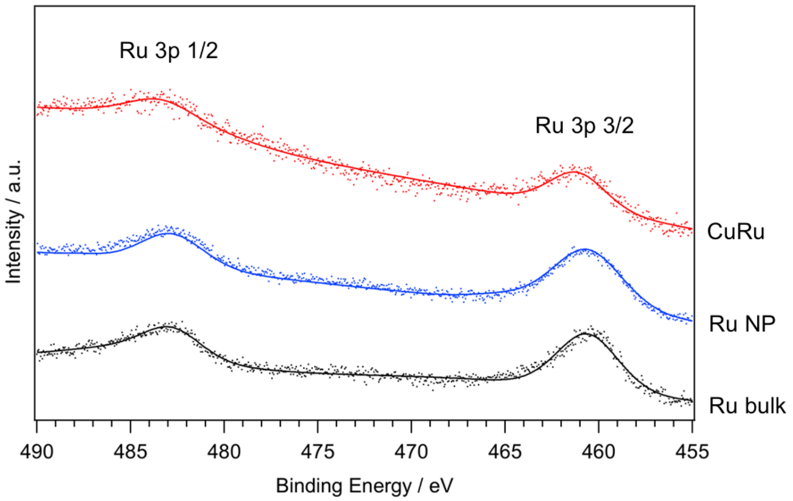

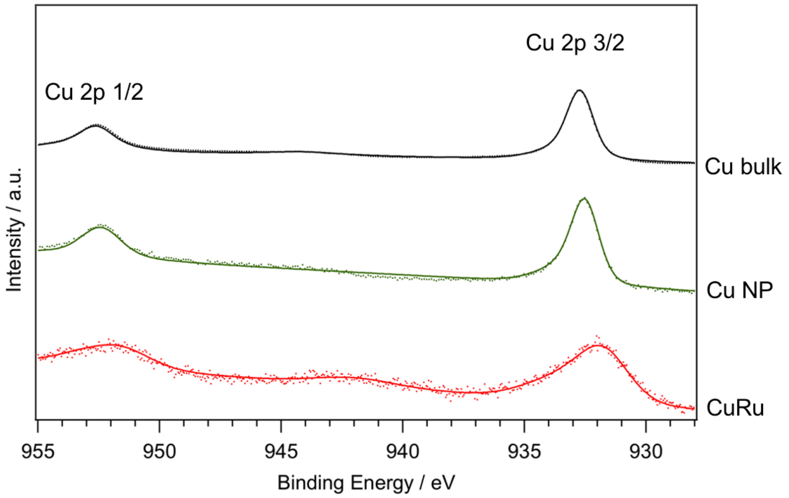

Fig. S32 The Ru 3p and Cu 2p core-level XPS of CuRu nanoparticles and reference samples. The weak satellite peak in Cu 2p binding energy of CuRu NPs was caused by surface oxidation during sample transfer.

Table S12 Binding energies of Ru 3p and Cu 2p.

Samples	BE(Ru $3p_{1/2}$)(eV)	BE(Ru $3p_{3/2}$)(eV)	BE(Cu $3p_{1/2}$)(eV)	BE(Cu $3p_{3/2}$)(eV)
**Ru bulk**	482.8	460.7		
**Ru NP**	482.7	460.6		
**CuRu**	483.4	461.3	951.9	932.0
**Cu bulk**			952.7	932.8
**Cu NP**			952.5	932.6

17. Thermal stability

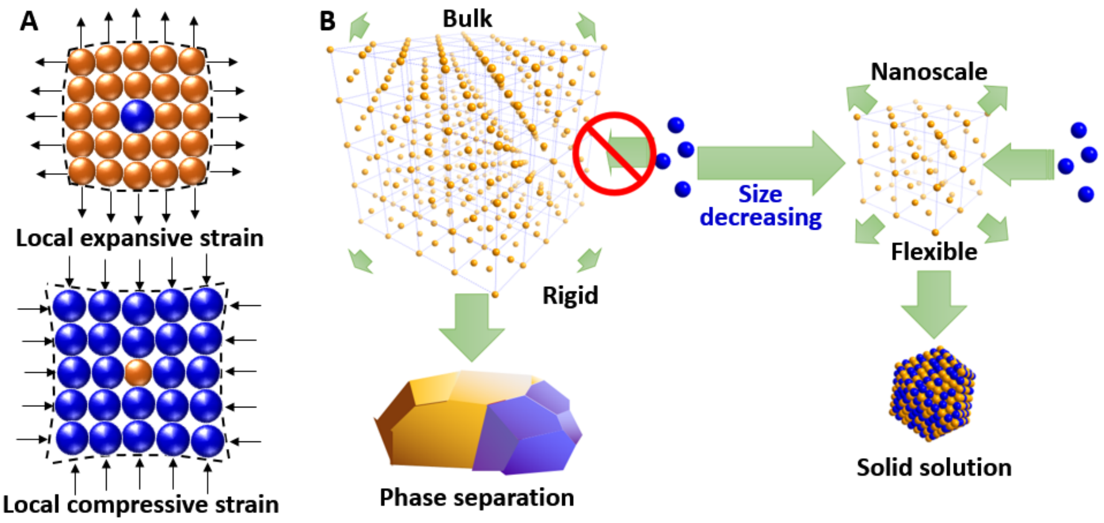

Fig. S33 (A) Local lattice strain from radius difference. (B) The proposed lattice relaxation mechanism for stabilization of solid-solution state by size decreasing.

18. Rietveld refinement Results on *in situ* synchrotron PXRD patterns at 773 K and select area analysis of STEM-EDX mapping images after thermal stability test.

Table S13 Structural parameters for $Cu_{0.5}Ru_{0.5}$ nanoparticles

T = 773 K, R_{wp} = 4.88%, GOF = 1.53

(a) fcc phase, space group: *Fm*-3*m*

Atom	*x* , *y*, *z*	Occupancy#
Ru	0, 0, 0	0.50
Cu	0, 0, 0	0.50

(b) hcp phase, space group: $P6_3/mmc$

Atom	*x* , *y*, *z*	Occupancy#
Ru	1/3, 2/3, 1/4	0.50
Cu	1/3, 2/3, 1/4	0.50

Occupancy was fixed.

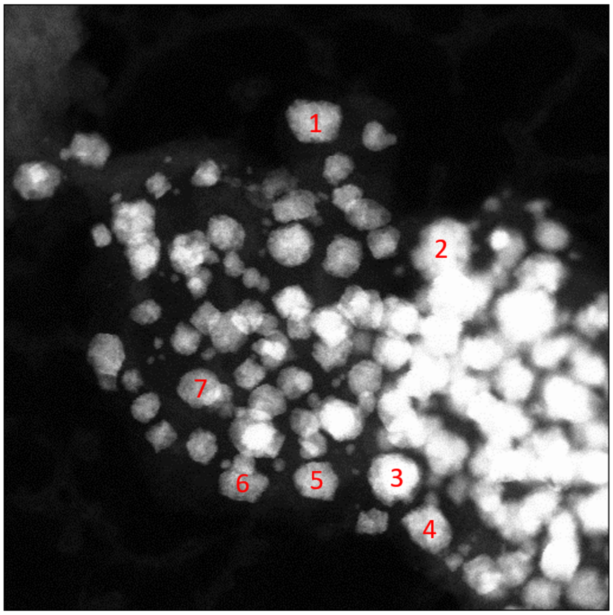

	Element Line	K-Factor	Wt.%	Wt.% Error	Atom %	Atom % Error
1	Cu K	1.340	36.92	±1.84	**48.21**	± 2.39
	Ru L	1.713	63.08	±3.42	**51.79**	± 2.80
2	Cu K	1.340	39.32	±1.51	**50.75**	± 1.93
	Ru L	1.713	63.68	±2.88	**49.25**	± 2.34
3	Cu K	1.340	38.21	±1.42	**49.58**	± 1.84
	Ru L	1.713	61.79	±2.71	**50.42**	± 2.21
4	Cu K	1.340	30.40	±1.84	**41.00**	± 2.48
	Ru L	1.713	69.60	±3.87	**59.00**	± 3.28
5	Cu K	1.340	36.36	±2.31	**47.60**	± 3.03
	Ru L	1.713	63.64	±4.43	**52.40**	± 3.63
6	Cu K	1.340	32.95	±2.10	**43.87**	± 2.77
	Ru L	1.713	67.05	±4.40	**56.13**	± 3.66
7	Cu K	1.340	34.83	±2.42	**45.94**	± 3.21
	Ru L	1.713	65.17	±4.74	**54.06**	± 3.93

Fig. S34 Select area analyses of each particle in STEM-EDX mapping of CuRu alloy NPs after heating at 773 K. The atomic ratio of Cu:Ru is nearly equal to 1:1, which is same to the nominal ratio. No difference was found before and after heating up to 773 K.

19. Proposed mechanism for high stability obtained from coreduction

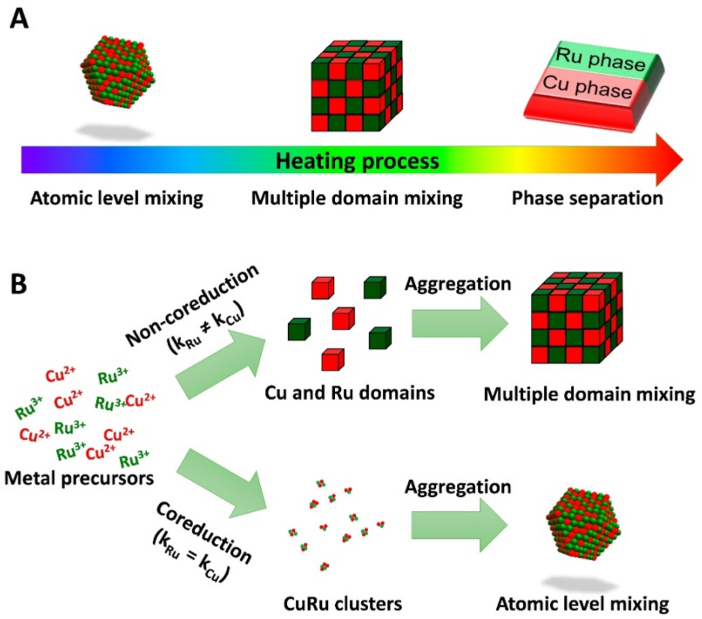

Fig. S35 (A) Schematic illusion for heating process of atomic level mixing alloy, via a multiple domain mixing state to phase separation state. (B) Schematic illusion for multiple domain mixing and atomic level mixing alloys obtained from non-coreduction (eg. rapid synthesis) and coreduction conditions, respectively.

Explanation to Fig. S35. In some synthetic process for bimetallic alloys, even the reduction velocities of each precursor are not same, by some techniques such as rapid reduction, when the reduction times of precursors is much shorter than diffusion time of metal atoms in solution, the random alloy structure could also be obtained. In that case, since the reduction velocities are not same, the random alloy structure is close to a multiple domain mixing structure shown in Fig. S35B, which is considered to be more unstable compared with atomic level mixing structured alloy obtained from well-optimized coreduction condition (Fig. S35A).

20. The TEM images of samples for exhaust purification test

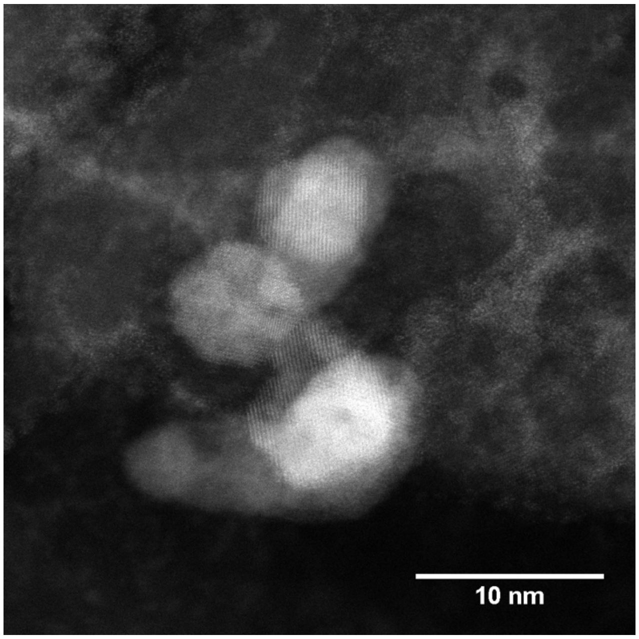

Fig. S36 HAADF-STEM image of CuRu solid solution supported on γ-Al_2O_3 for exhaust purification test.

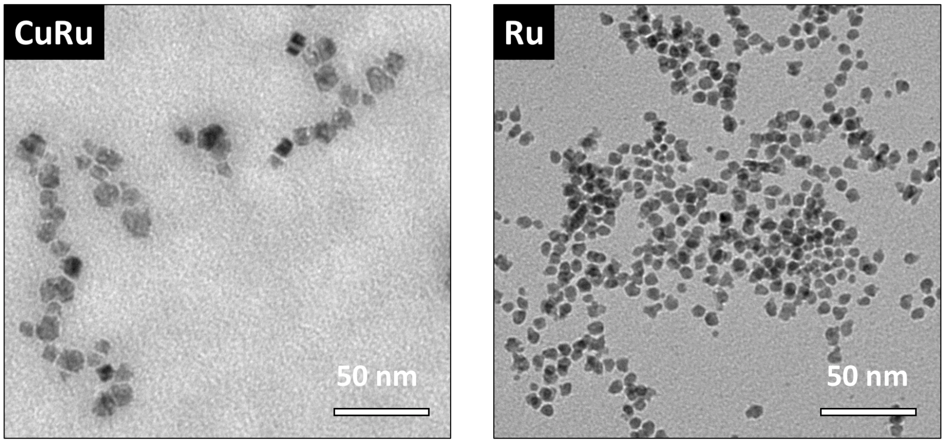

Fig. S37 TEM images of CuRu solid solution and Ru nanoparticles for exhaust purification test. The mean diameter of CuRu solid solution and Ru nanoparticles were estimated to be 9.2 ± 2.5 nm and 7.7±1.4 nm, respectively.

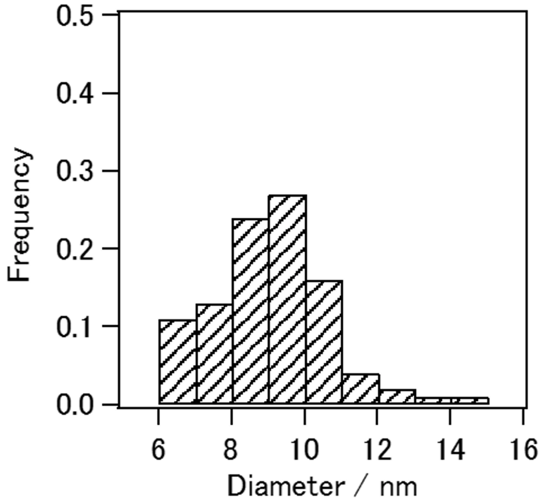

Fig. S38 Size distribution for CuRu solid solution supported on γ-Al_2O_3.

21. Durability test

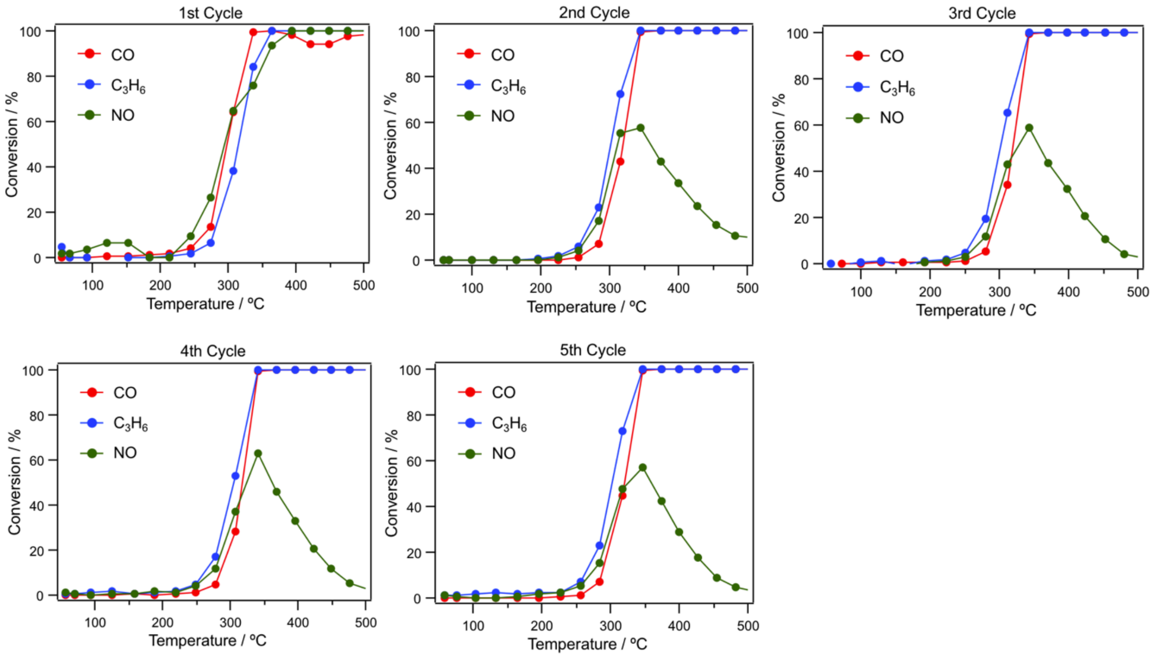

Fig. S39 Five cycles three-way catalytic durability test for CuRu solid solution.

References

1. P. Vanýsek, *CRC Handbook of Chemistry and Physics, 84th Edition*, (Eds.: D. R. Lide), Taylor & Francis, Boca Raton, Florida, USA 2003, 1217.
2. K. H. Park, S. H. Im and O O. Park, *Nanotechnology,* 2011, **22**, 045602.
